# Genome-wide association mapping and genomic prediction for adult stage sclerotinia stem rot resistance in *Brassica napus* (L) under field environments

**DOI:** 10.1038/s41598-021-01272-9

**Published:** 2021-11-05

**Authors:** Jayanta Roy, T. M. Shaikh, Luis del Río Mendoza, Shakil Hosain, Venkat Chapara, Mukhlesur Rahman

**Affiliations:** 1 Department of Plant Sciences, North Dakota State University, Fargo, ND 58108 USA; 2 Department of Plant Pathology, North Dakota State University, Fargo, ND 58108 USA; 3 Langdon Extension Research Extension Center, North Dakota State University, Langdon, ND 58249 USA

**Keywords:** Computational biology and bioinformatics, Genetics, Plant sciences

## Abstract

Sclerotinia stem rot (SSR) is a fungal disease of rapeseed/canola that causes significant seed yield losses and reduces its oil content and quality. In the present study, the reaction of 187 diverse canola genotypes to SSR was characterized at full flowering stage using the agar plug to stem inoculation method in four environments. Genome-wide association study (GWAS) using three different algorithms identified 133 significant SNPs corresponding with 123 loci for disease traits like stem lesion length (LL), lesion width (LW), and plant mortality at 14 (PM_14D) and 21 (PM_21D) days. The explained phenotypic variation of these SNPs ranged from 3.6 to 12.1%. Nineteen significant SNPs were detected in two or more environments, disease traits with at least two GWAS algorithms. The strong correlations observed between LL and other three disease traits evaluated, suggest they could be used as proxies for SSR resistance phenotyping. Sixty-nine candidate genes associated with disease resistance mechanisms were identified. Genomic prediction (GP) analysis with all the four traits employing genome-wide markers resulted in 0.41–0.64 predictive ability depending on the model specifications. The highest predictive ability for PM_21D with three models was about 0.64. From our study, the identified resistant genotypes and stable significant SNP markers will serve as a valuable resource for future SSR resistance breeding. Our study also suggests that genomic selection holds promise for accelerating canola breeding progress by enabling breeders to select SSR resistance genotypes at the early stage by reducing the need to phenotype large numbers of genotypes.

## Introduction

*Sclerotinia sclerotiorum* (Lib.) de Bary is a devastating non-host specific, necrotrophic and ubiquitous plant pathogenic fungus that infects at least 408 plant species including economically important dicotyledonous crops such as oilseed rape, edible dry bean, soybean, sunflower, pea, chickpea, lentils, and different types of vegetables and some monocotyledonous crops such as tulip and onion^1,2^. The disease caused by this pathogen in rapeseed/canola is commonly referred to as sclerotinia stem rot (SSR), and it significantly limits rapeseed yield/production worldwide. This disease imposes 10–20% yield loss per year in China, but the loss could be up to 80% in severely infected fields^3^. In the United States, where every percentage unit of incidence reduces on average 0.5% of canola potential yields^4^, the annual seed loss due to this pathogen attack has been estimated at about $24 million^5^. Besides seed yield loss, the disease also reduces the oil content and makes changes in the fatty acid profile of affected plants that reduces oil quality^6–8^.

The pathogen survives in the soil up to 8–10 years by producing long-lived melanized resting structures called sclerotia^9,10^. Under favorable conditions (i.e., moderate to high moisture and moderate soil temperatures) the sclerotia germinate carpogenically. Airborne ascospores released during the day^11^ and start infection by colonizing on senescent petals. In the presence of free moisture, ascospores germinate and move from petals to leaves^12^ and main stems, where forming lesions may completely girdle the stem and cause death of plant^13^.

Currently, rapeseed/canola growers depend primarily on use of crop rotation with non-host crop species and prophylactic fungicide applications for SSR management due to the unavailability of SSR resistant varieties^14^. However, long-term persistent survivability of the sclerotia in the soil and wider host range of the fungus makes crop rotations less effective. Moreover, properly timing fungicide applications to manage the disease is a difficult task to achieve and adds additional input cost and has a negative impact on the environment. Therefore, breeding for disease resistant rapeseed/canola varieties would be an economically feasible, more efficient, and environmentally friendly option. Hereafter, it is crucial to study different germplasm from diverse regions in order to unravel the nature of durable genetic resistance and identify responsible genes for the resistance to *S. sclerotiorum*. Subsequently, such identified SSR resistance genes can be introduced into high performing elite canola cultivars which ultimately diminish the dependence of the canola growers on cultural practices and use of fungicides and making canola production more profitable.

Unfortunately, breeding for SSR resistance is a challenging task as sources of complete resistance to the disease have not been identified in *Brassica napus* and its close relatives in more than three decades of investigation^15,16^. Instead, few germplasms with partial resistance have been identified and utilized in the SSR resistance breeding. Several genetic studies have shown that the mode of SSR resistance is quantitatively inherited with additive effect and have medium to high heritability^17–20^. To date, genetic mapping studies has been carried out to identify the sclerotinia resistance loci in multiple bi-parental mapping populations developed from crosses between resistant and susceptible parents^15,16,20–24^. QTL mapping using bi-parental populations have detected several QTLs for SSR resistance, and the majority of them located on chromosomes A09, C02, and C06. However, in very few instances common markers have been detected in different mapping populations^24^, which makes it difficult to identify the overlapping QTLs. The availability of the *B. napus* reference genome sequence^25^ offers an opportunity to determine the physical location of the previously identified QTLs by aligning the QTL primers with the *B. napus* genome. Integration and comparative analyses of the previously identified resistance QTLs from various mapping studies with the reference genome sequence have detected conserved QTLs on chromosome A9 (22.5–27.5 Mb) and C6 (29.5–36.1 Mb)^26^. Despite these successes, no fine mapping or map-based cloning for sclerotinia resistance gene has been reported so far, which ultimately circumvents the utilization of identified QTL in the SSR resistance breeding strategy. To date, all identified sclerotinia resistance QTLs only explained a small portion of phenotypic variance and few QTLs could be detected in different populations, different growth stages, or different screening methods. Moreover, bi-parental QTL mapping strategy suffers from low allelic diversity since only two allelic effects were evaluated for a single locus and limited recombination events which leads to the lower mapping resolution^27^. Apart from bi-parental linkage mapping strategy, genome-wide association study (GWAS) uses natural population originated from non-cross derived lines which offer extensive historical recombination events, shorted linkage disequilibrium (LD) segments thus provides a promising opportunity of having high mapping resolution for the marker-trait-association^28^.

Recently, the dramatic reduction of sequencing cost and quick turn-around time has led to the development of genome-wide dense molecular markers, which further accelerated the application of GWAS and genomic selection (GS) towards the genetic improvement of complex traits. To highlight the effectiveness of GWAS to improve the enhanced SSR resistance and marker-assisted selection, to date, only a few genetic mapping studies through GWAS for SSR on rapeseed/canola have been reported. Gyawali et al.^29^ used 84 simple sequence repeat markers to conduct a GWAS study using 152 *B. napus* accessions at the flowering stage in a controlled environment and identified 34 significantly associated loci of which 21 alleles contributed to the SSR resistance. Wei et al.^18^ used 30,932 SNP markers to conduct a GWAS study and detected five significant associations on A8, and twelve on C6 using detached stem inoculation method. A total of 26 SNPs associated with SSR resistance were identified on chromosome C4, C6, and C8 from a field study based on detached stem inoculation assay and the genotyping data of 25,573 SNPs by Wu et al.^19^. However, the effectiveness of GWAS may be limited in detecting common alleles with very small effects, as well as rare variants with small effect^30^. Similar to GWAS, the GS is performed by employing genome-wide markers distributed throughout the genome. GWAS detects significant SNP-trait associations that account only a small portion of phenotypic variance, indicating that there is a significant amount of genetic information that could be captured with a whole genome modeling. Contrary to GWAS, GS has emerged as a promising genomics-assisted techniques that uses all the molecular markers information and phenotype data of the training population to develop statistical models that predict genomic estimated breeding values (GEBVs) in testing individuals based only on the genotype information^31,32^. A number of GS studies have been reported in several crops such as wheat^33–35^, and maize^36–38^ in the past 10 years. The potential of GS in rapeseed/canola was investigated for various agronomic traits including resistance to blackleg disease^39,40^ and concluded as a promising tool for rapeseed breeding. However, the application of genomic prediction for SSR resistance in rapeseed/canola has been limited to date^18,41^. Recently, Derbyshire et al.^41^ implemented GP on adult plant SSR resistance in *B. napus* and reported that the GS can be used for the improvement of *S. sclerotiorum* resistance.

The objectives of the study were (i) to identify SSR resistant genotypes, (ii) dissect the genetic architecture of SSR resistance, (iii) identify the genomic regions, marker-trait-associations (MTAs), and putative candidate genes conferring SSR resistance, (iv) to explore and evaluate the effectiveness of genomic prediction (GP) for selection of genotypes for SSR resistance.

## Materials and methods

### Plant materials and experimental design

A panel of 187 diverse spring and semi-winter *B. napus* germplasm accessions and breeding lines originating from 17 countries in the world were collected from North Central Regional Plant Introduction Station (NCRPIS), Ames, Iowa, USA and North Dakota State University (NDSU) (Supplementary Table S1). Both NCRPIS and NDSU are public institutions that comply with all necessary regulations to use the seed materials for research and development purposes. The panel was planted in North Dakota State University Agricultural Experiment Station at Carrington, and Langdon in 2019; Carrington and Osnabrock (similar weather conditions and near to Langdon Research Station) in 2020. All the field experiments were carried out using a randomized complete block design with three replications. Each line was grown in six-rows plots (1.5 m × 1.2 m) with 40 plants per row. Rows in each plot were distanced 25 cm apart. The field management was done essentially using regular breeding practices. Two commercially available spring canola hybrid cultivars, Pioneer 45S51 and Pioneer 45S56, as resistant checks, and publicly available cultivar Westar as a susceptible check were used in the study.

### Disease phenotypic evaluation and plant phenotypic measurements

**Figure 1:**
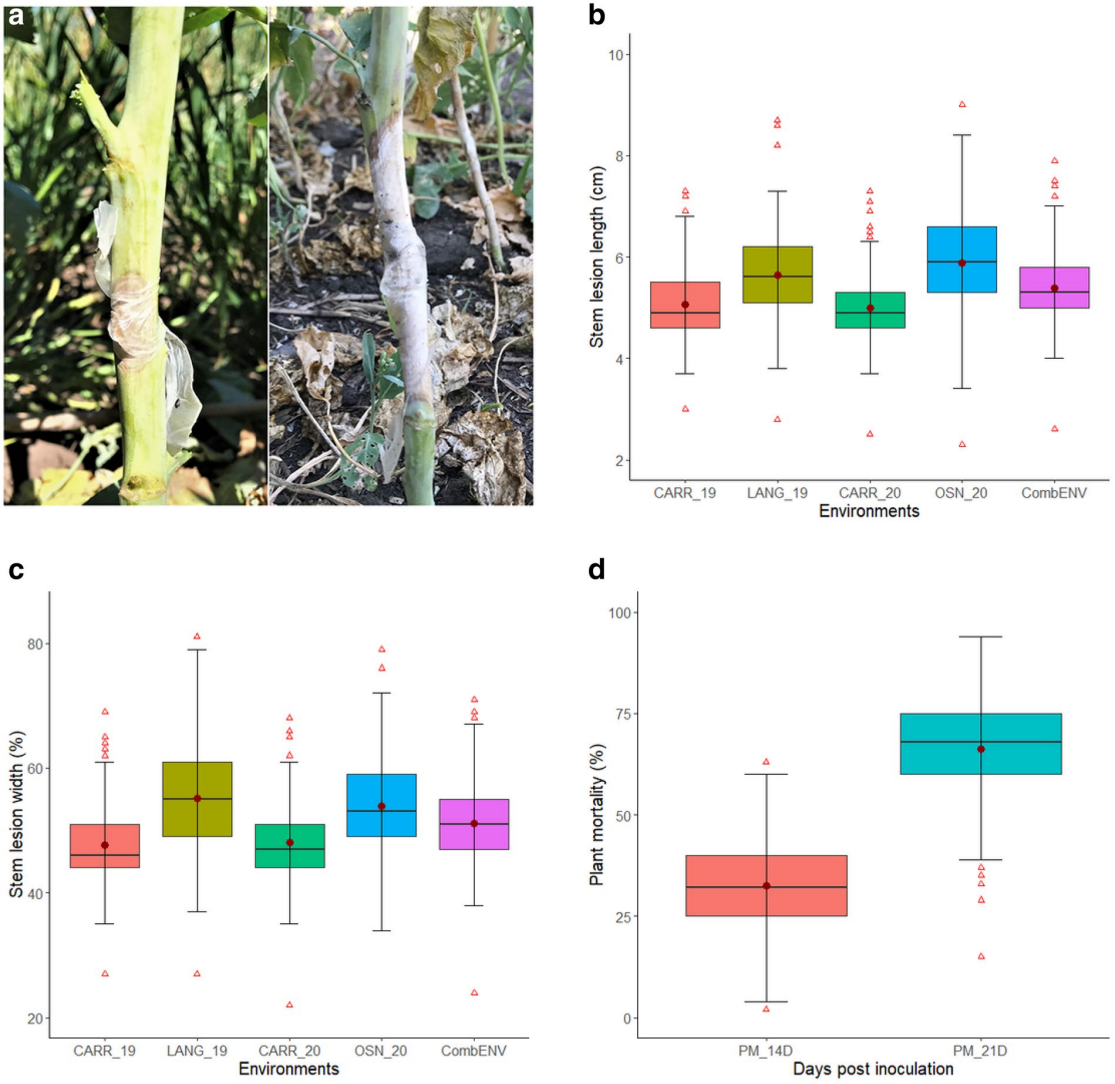
Average disease phenotypic characteristics of 187 *Brassica napus* genotypes evaluated at Carrington in 2019 (CARR_19) and 2020 (CARR_20), Langdon in 2019 (LANG_19), Osnabrock in 2020 (OSN_20) and combined across four environments (CombENV), North Dakota. Sclerotinia stem rot lesions on the most resistant (left) and susceptible (right) genotypes seven days post inoculation (dpi) (**a**). Boxplots of BLUEs (Best Linear Unbiased Estimates) values for lesion length (cm) measured 7 dpi (**b**); lesion width (%) measured 7 dpi (**c**); and percentages of plant mortality estimated 14 and 21 dpi (**d**). BLUEs for plant mortality are averages of all environments. Box edges represent the upper and lower quartile with median value shown as a bold line in the middle of each box. Mean values are represented by red circle, and the upper and lower whiskers represent the extreme values.

In this study, we used *S. sclerotiorum* isolate WM031 for all inoculations. This isolate has been used in previous studies because of its high virulence to canola^24^. The isolate was cultured on autoclaved potato dextrose agar medium (24 gL^−1^ potato dextrose broth and 1.5 gL^−1^ agar) at 22–24 ℃ for 48 h. The canola plants were inoculated at full flowering stage. The main stem of eight arbitrarily selected plants from each row were inoculated by placing a 7 mm agar plug containing actively growing hyphal tips approximately at a height of 40–50 cm above the ground (Fig. 1a). Each plug had the hyphal side facing the epidermis of the plant and was held in place by wrapping it to the stem with parafilm to ensure close contact between the pathogen and the stem surface and to maintain humidity. The lesion length (LL) on the main stem was measured at 7 days post inoculation (dpi) using a measuring scale. Stem lesion width (LW) with a visual estimation of the percentage of the main stem were girdled by the lesion was also collected at 7 dpi. The status of inoculated plants, dead/alive was recorded at 14 and 21 dpi and used to calculate percentage of plant mortality, therefore designated as PM_14D and PM_21D, respectively. Eight inoculated plants per replication of each genotype were sampled for disease evaluation, which resulted in a total about 96 (8 plants × 3 replications × 4 environments) plants evaluation for each accession throughout the study.

Flowering time (FT) is an important developmental stage of the flowering plants in which they switch from the vegetative stage to the reproductive stage and it may have a role in the plant pathogen interactions^42^. Therefore, data on days to flowering (DF) was recorded from days of seeding to flowering (when 50% of the plants in each replication of each genotype started flowering) during the year of 2019 and 2020 in both locations. In addition to FT, stem diameter (SD) and stem internode length (IL) may have an effect on SSR disease prevalence on *B. napus*. Therefore, to investigate its effect on SSR resistance, we collected the data on plant SD and IL of the inoculated plant keeping in mind to have the equal number of plants in each plot considering the plant development stage. Data on stem diameter (SD) in both years was measured using a Vernier caliper from the inoculated internodes slightly above where the inoculum was applied at 7 dpi. The internode length (IL) was taken using a measuring scale from the inoculated internode stems at 7 dpi from all the studied environments.

### Statistical analyses

The four field experiments were designated as 2019 Carrington (CARR_19), 2019 Langdon (LANG_19), 2020 Carrington (CARR_20), and 2020 Osnabrock (OSN_20), were analyzed individually and as a combined set with SAS 9.4 (SAS Institute Inc., USA). The best linear unbiased estimates (BLUEs) were calculated within single environment analysis and combined analysis across environments (combENV). For the combined analysis across all environments (combENV), homogeneity of variance was determined by dividing the environment with the highest error mean squares by the environment with lowest error mean squares. If the calculated ratio was less than tenfold, then the data from all environments were combined^43,44^. For the environment-wise analyses, genotypes were considered as fixed effects, and replications as random effects. Variance components were used to calculate the broad-sense heritability (*H*^2^) as

$${H}^{2}=\frac{{\sigma }_{g}^{2}}{{\sigma }_{g}^{2}+{\sigma }_{e}^{2}}$$

For the combENV, a mixed linear model was implemented considering genotypes as fixed effects, environment, replication within the environment, and genotype-by-environment interaction as random effects. The broad-sense heritability (*H*^2^) for each trait was computed for the combENV set as

$${H}^{2}=\frac{{\sigma }_{g}^{2}}{{\sigma }_{g}^{2}+\frac{{\sigma }_{ge}^{2}}{n}+\frac{{\sigma }_{e}^{2}}{nr}}$$

where *σ*^2^_*g*_ is the genotypic variance, *σ*^2^_*ge*_ is the genotype by environment variance, *σ*^2^_*e*_ is the residual error variance, *n* is the number of environments, and *r* is the number of replications per environment. Environment-wise and combENV BLUEs for LL and LW phenotypic trait, and only combENV BLUEs for PM_14D and PM_21D were used to perform GWAS analyses. Correlations among all traits were performed by calculating Pearson correlation coefficients in SAS 9.4. To determine whether the collected data was normally distributed, the Shapiro–Wilk test was conducted for all traits in both single environment and a combined dataset. Simple linear regression analysis was performed with stem LL, LW, PM_14D and PM_21D as dependent variables and flowering time as independent variable in R^45^ (R Development Core Team). The same analysis was performed to examine the relationship between SD and IL as independent variables and stem LL, LW, PM_14D and PM_21D as dependent variables.

### Genotyping

Fresh young leaf tissues were harvested from each germplasm and lyophilized at -80̊ C until used. The total genomic DNA was extracted from the lyophilized tissues using Qiagene DNeasy kit (Qiagen, CA, US) following the manufacturer’s protocol. Then the extracted DNA was quantified using NanoDrop 2000/2000c Spectrophotometer (Thermofisher Scientific) and optimized to get the same concentration of DNA. The GBS library was prepared using the *ApekI* enzyme following the protocol described by Elshire et al.^46^. The prepared GBS library was sent to the University of Texas Southwestern Medical Center, Dallas, Texas, USA for DNA sequencing using Illumina HiSeq 2500 sequencer. Single end sequencing reads were mapped to the *B. napus* ‘ZS11’ reference genome^47^ using Bowtie 2 (version 2.3.0) alignment tool^48^ with the default parameters. SNP calling was done using TASSEL 5 GBSv2 pipeline^49^ and 497,336 unfiltered SNPs were identified. High quality SNP were identified through filtering with VCFtools^50^ with the following criteria: minor allele frequency (MAF) ≥ 0.05, missing values (max-missing) ≤ 25%, depth (minDP) ≥ 3 and physical distance (thin) ≤ 500 bp. As canola is a self-pollinating crop, more than 25% heterozygous SNP were removed using TASSEL^51^. The SNPs that were located outside of the chromosomes (i.e. unknown position), were removed. Thus, a total of 38,510 high quality SNPs were retained. For the present study, we utilized the polymorphic SNP markers data with minor allele frequencies greater than 0.05 on 187 genotypes.

### Maker-trait-associations

Marker-trait-association analyses were implemented in the GAPIT R package (version 3.0)^52^, and GEMMA-MLM^53^. In the GAPIT analysis, we computed principal component (PC) analysis for accounting population structure using *prcomp ()* function^54^ in R and kinship (K) matrix by VanRaden method^55^ for relationships among individual, both using molecular marker data. The first four PCs were used for model-based clustering analysis to determine the subpopulations using the Mclust package in R. The first four PCA as a population structure and kinship matrix were incorporated in the GWAS models to control false-positives. The single locus mixed linear model (MLM)^56^, and the multi-locus model fixed and random model circulating probability unification (FarmCPU)^57^ were implemented in GAPIT. Additionally, the identified significant MTAs were verified by performing GWAS analyses in another commonly used software i.e. GEMMA-MLM (version 0.98.1) through the execution of the command: “gemma -g [genotype file] -a [genotype annotation file] -p [phenotype] -c [first 4PCA] -k [kinship/centered relatedness matrix] -o [output file]. For this analysis, the incorporated first four PCs were the same that we used for FarmCPU, and MLM. The kinship matrix was generated using the centered relatedness procedure in GEMMA, used as a random effect variable in the random model. *P-wald* test (the improved calibrated *P* value in GEMMA) was calculated for the given model. The significant threshold of *P* value for the association between SNPs and traits were determined following the method proposed by Li and Ji^58^. The effective number of independent tests (M_eff_) among the used 25,809 SNPs were determined by calculating the correlation matrix and eigenvalue decomposition. Then the Bonferroni correction was applied based on the effective number of independent tests (loci). The effective number of independent tests was estimated as 127, thus by applying genome-wide type I error rate at *α* = 0.05, the determined significant threshold of *P* = 0.05/127 = 0.0004 or − log_10_ (*P*) = 3.4. Therefore, the significant threshold value for the association between SNP and traits were determined by − log_10_ (*P*) ≥ 3.4, which is equivalent to *P* ≤ 0.0004, for FarmCPU, MLM, and GEMMA-MLM. The SNPs detected by at least two models in at least one environment were declared as significant and considered as relatively stable significant SNP. To identify the common significant SNP markers present in more than one environment, a threshold value of − log_10_ (*P*) ≥ 3.00 was used, only when those SNPs that had a lower association threshold (*P* ≤ 0.0004) in one environment were considered common. Manhattan plot and *P value* distributions by plotting the observed *P values* against expected *P values* shown in Q–Q plots were created using the mhplot package in R language.

### Candidate gene identification

Candidate genes were searched for those significant SNPs that were detected in more than two environments, and two or more GWAS models. Genes present within 50 kb upstream and downstream of the significant markers were considered as candidate genes based on the genome and the gene models by ‘ZS11’ reference genome sequence^47^. Protein sequences from the gene models were blasted against TAIR 10 protein database to determine the gene annotation. Genes associated with defense response were identified based on the Gene Ontology terms (GO terms) from TAIR website and gene functions found in the previous literature, TAIR 10 and Uniport-KB.

### Genomic prediction

The genomic prediction models were constructed with the following formula:

$$y=\mu +X\beta +\varepsilon$$

where *y* is the vector of the phenotypic observations, *µ* is the grand mean, *X* is the marker genotype matrix, *β* is the estimated random additive marker effect, and *e* is the residual error term. Three GS models i.e. ridge regression best linear unbiased prediction rrBLUP^59^ and two Bayesian models: Bayes C^60^, and Bayesian Ridge Regression (BRR)^31^ were used for implementing genomic prediction. All models were analyzed in R language. The GP model rrBLUP, and two Bayesian models were constructed using the package “rrBLUP”^59^, and BGLR (version 4.0.4)^61^ package, respectively. For all the analyses, Bayesian models were performed for 5000 Monte Carlo Markov chain iterations with a 1000 burn-iterations. The environment-wise BLUEs of LL, LW, and the combENV BLUEs of LL, LW, PM_14D and PM_21D were used as phenotypic values for subsequent GP analyses. All SNP markers (25,809 SNPs) distributed in the whole genome were employed in the GP. A fivefold cross validation (150 individuals as training population and 37 individuals as validation individuals) with 100 iterations or 100 rounds of random sampling were implemented to assess the accuracy and predictive ability of the GP model for each predicting phenotypic traits with the validation populations. Predictive ability was defined as the correlation (Pearson’s *r*) between genomic estimated breeding values (GEBVs) and the observed phenotypic value. The measure of prediction accuracy is the correlation between GEBV and an estimate of true breeding value. However, true breeding values for this trait are typically unknown. Therefore, the prediction accuracy was indirectly estimated by dividing the correlation between GEBV and observed phenotypic value by an estimate of square root of heritability ($\surd {h}^{2}$)^41,62^. Thus, the prediction accuracy of each model was estimated by dividing the mean predictive ability by square root of heritability ($\surd$*h*^2^).

### Genomic heritability

Genomic heritability (narrow-sense heritability, *h*^2^) was estimated for the combined datasets of all the evaluated traits for each model using all the markers. The additive variance components (*Va*) and the residual variance components (*Ve*) were estimated with *mixed.solve* function in rrBLUP for the rrBLUP model^59^. Narrow-sense heritability (*h*^2^) was assessed by dividing the additive genetic variance (*Va*) by the total variance estimate (sum of additive variance and the residual variance). For the Bayesian models, *h*^2^ was estimated by taking the average proportion of variance explained by the regression of phenotypes on molecular markers as described by de los Campos et al.^63^ (https://github.com/gdlc/BGLR-R/blob/master/inst/md/heritability.md).

## Results

### Phenotypic evaluations

A continuous and broad range of reactions to inoculation with *S. sclerotiorum* was observed among the 187 *B. napus* accessions in this study (Table 1, Fig. 1a–d). The main stems LL of the genotypes at 7 dpi varied from 2.3 to 9.0 cm and LW ranged from 19.3 to 81.4% among the studied environments (Table 1, Fig. 1b,c). In the CARR_19, the main stems LL ranged from 3.0 to 7.3 cm with the mean of 5.1 cm, whereas LANG_19, CARR_20, and OSN_20 had a range (mean) of 2.8–8.7 cm (5.6 cm), 2.5–7.3 cm (5.0 cm), 2.3–9.0 cm (5.9 cm), respectively (Table 1, Fig. 1b). In the case of stem LW, the CARR_19, LANG_19, CARR_20, and OSN_20 had a range (mean) of 26.9–70.3% (47.7%), 26.7–81.4% (55.1%), 21.9–68.0% (48.1%), and 19.3–78.6% (53.6%), respectively (Table 1, Fig. 1c). The mean LL (5.9 cm) was the highest in OSN_20, and the lowest (5.0 cm) was recorded in CARR_20, whereas the highest overall mean LW (55.1%) was observed in LANG_19 followed by the lowest mean (47.7%) in both CARR_19 environments (Table 1, Fig. 1b,c). The resulting BLUEs for PM_14D and PM_21D for SSR scores across all (combENV) environments ranged from 2 to 63% with an average of 33%, and from 16 to 94% with a mean of 66%, respectively (Table 1, Fig. 1d). The diversity of phenotypic responses observed in this study is consistent with observations made by other researchers^18–20,22,24^ and reinforce the notion that resistance to sclerotinia infections is quantitatively inherited and controlled by multiple genes. The combENV BLUEs of the top five promising source of resistance ranged between 2.6 and 4.2 cm for LL, from 23.7 to 40.6% for LW, and had between 2 and 10% PM_14D, and between 16 and 37% PM_21D. These ranges were smaller than the respective phenotypic responses observed on the resistant checks “Pioneer 45S51” 5.2 cm, 51.9%, 32%, and 66% for LL, LW, PM_14D, and PM_21D, respectively, and “Pioneer 45S56” 5.2 cm, 49.5%, 31%, and 62% for LL, LW, PM_14D, and PM_21D, respectively, and susceptible check “Westar” 6.8 cm, 65%, 63%, and 94% for LL, LW, PM_14D, and PM_21D, respectively (Supplementary Table S1). Therefore, these promising genotypes will serve as a valuable resource to transfer resistance gene into the elite canola cultivars to develop SSR resistant cultivars for the growers. A two-way analysis of variance (ANOVA) indicated that genotype, environment, and their interaction had significant effects (*P* ≤ 0.001) on both LL and LW for stem resistance. Similar results were obtained from the ANOVA analysis of the combENV sets for PM_14D, and PM_21D with the exception of the interaction between genotype and environment on PM_21D, which was not-significant (*P* ≤ 0.05) (Supplementary Table S2). The environment-wise heritability on phenotypic mean basis for LL ranged between 0.64 and 0.78, while heritability for LW ranged between 0.56 and 0.72. Combined across all environments, high broad-sense heritability 0.88 and 0.86 was observed for LL and LW, respectively (Table 1).

**Table 1:** Phenotypic variation obtained through BLUEs in the response of 187 *Brassica napus* genotypes sclerotinia stem rot.

Traits	Env.	Unit	Min	Mean	Max	Range	CV	Shapiro–Wilk test *p* value	*H*^2^ (family mean basis)
LL	CARR_19	cm	3.0	5.1	7.3	4.3	24.8	0.0001	0.70
	LANG_19	cm	2.8	5.6	8.7	5.9	26.6	0.0152	0.64
	CARR_20	cm	2.5	5.0	7.3	4.8	22.7	0.0000	0.78
	OSN_20	cm	2.3	5.9	9.0	6.7	26.8	0.2339	0.70
	CombENV	cm	2.6	5.4	7.9	5.3	25.6	0.0002	0.88
LW	CARR_19	%	26.9	47.7	70.3	43.4	24.9	0.0006	0.65
	LANG_19	%	26.7	55.1	81.4	54.7	28.8	0.6422	0.56
	CARR_20	%	21.9	48.1	68.0	46.1	25.4	0.0007	0.72
	OSN_20	%	19.3	53.6	78.6	59.3	29.1	0.0694	0.63
	CombENV	%	23.7	51.2	71.3	47.6	27.5	0.0077	0.86
PM_14D	CombENV	%	2.4	32.6	63.1	60.7	53.3	0.7399	0.90
PM_21D	CombENV	%	15.5	66.2	94.0	78.5	26.4	0.0002	0.96
DF	CARR_19	days	41.3	50.7	79.3	38.0	3.7	0.0000	0.98
	LANG_19	days	37.3	46.7	77.3	40.0	4.0	0.0000	0.99
	CARR_20	days	43.0	57.6	87.0	44.0	3.8	0.0000	0.98
	OSN_20	days	42.7	54.5	88.0	45.3	6.4	0.0000	0.95
	CombENV	days	41.1	51.9	82.3	41.2	4.7	0.0000	0.98
SD	CARR_19	mm	4.4	7.01	12.0	7.6	31.2	0.0022	0.73
	LANG_19	mm	4.5	6.9	10.2	5.7	21.9	0.1440	0.65
	CARR_20	mm	4.0	6.9	11.6	7.6	23.7	0.0007	0.90
	OSN_20	mm	5.1	7.5	12.9	7.8	29.4	0.0001	0.73
	CombENV	mm	4.9	7.1	9.9	5.0	27.1	0.0032	0.88
IL	CARR_19	cm	7.8	11.6	14.5	6.7	17.9	0.1622	0.67
	LANG_19	cm	8.1	12.0	15.4	7.3	19.1	0.0002	0.77
	CARR_20	cm	8.4	10.8	18.9	10.5	28.3	0.0000	0.55
	OSN_20	cm	7.7	12.6	20.4	12.7	25.9	0.0139	0.75
	CombENV	cm	8.6	11.7	14.9	6.3	23.1	0.0922	0.79

*LL* lesion length measured 7 days post inoculation (dpi), *LW* lesion width measured 7 dpi, *Env* environments, Carrington 2019 (CARR_19) and 2020 (CARR_20), Langdon 2019 (LANG_19), Osnabrock 2020 (OSN_20), combined across all environments (CombENV), *Min* minimum, *Max* maximum value, *CV* coefficient of variation, *Shapiro–Wilk test* test for normality.

Plant phenotypic variables measured on the 187 genotypes in all environments displayed a wide variation. DF values ranged from 37 to 88 days and had a coefficient of variation (CV) ranging from 3.7 to 6.4 (Table 1). IL varied from 7.7 to 20.4 cm with CV between 17.9 and 28.3, whereas SD ranged from 4.0 to 12.9 mm with a CV ranging from 21.9 to 31.2 (Table 1). The frequency distributions of phenotypic values for each trait are presented in the Supplementary Fig. S1.

### Correlation among internode length, stem diameter, lesion length, and lesion width

**Figure 2:**
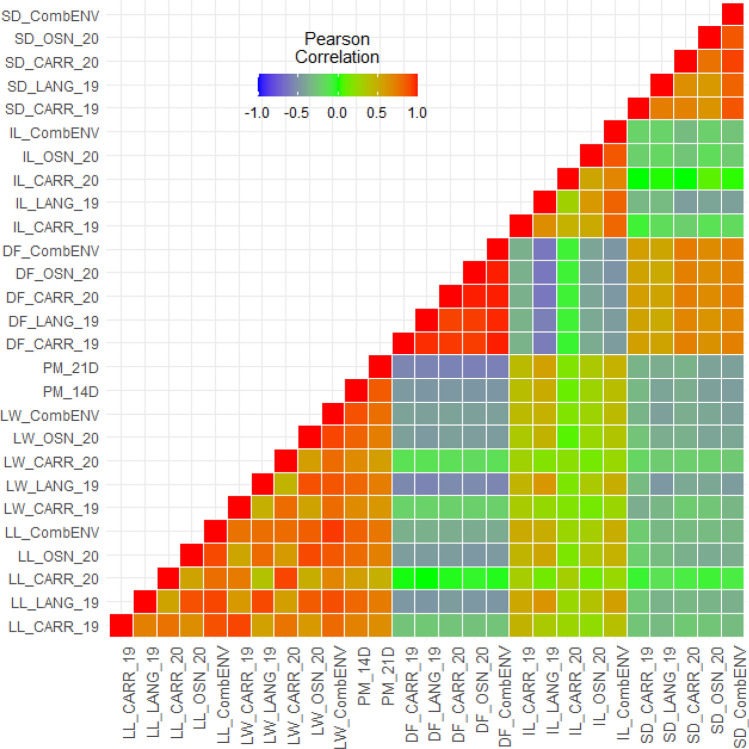
Correlation heatmap for different sclerotinia stem rot phenotypic traits in four environments and the combined dataset across environments. Traits: DF = days to flowering; IL = internode length; LL = lesion length, LW = lesion width, PM_14D = plant mortality at 14 days post inoculation (dpi); PM_21D = plant mortality at 21 dpi; SD = stem diameter. Environments: Carrington 2019 (CARR_19) and 2020 (CARR_20), Langdon 2019 (LANG_19), Osnabrock 2020 (OSN_20), combined across all environments (CombENV). Plant mortality are averages of all environments.

**Figure 3:**
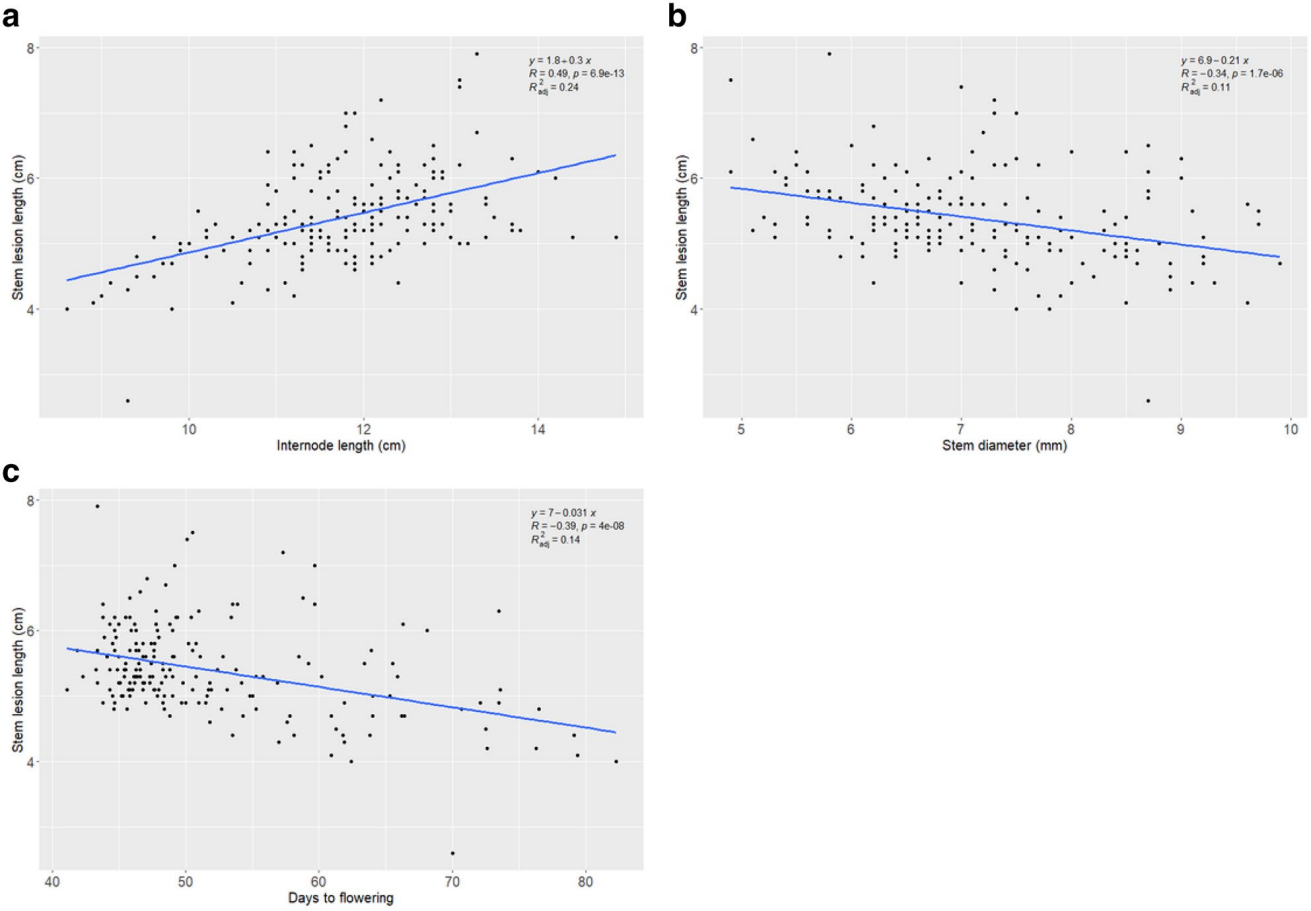
Regression analysis of SSR resistance in respect to (**a**) stem lesion length and internode length, (**b**) stem lesion length and stem diameter, and (**c**) stem lesion length and days to flowering. *R* is Pearson’s correlation coefficient between the two traits*, R*^2^_*ad*j_ is the coefficient of determination.

To determine the effects of IL and SD on the reaction of genotypes to SSR stem resistance in respect to stem LL, LW, PM_14D and PM_21D, efforts were made to maintain homogenous plant densities in every row. We found that combENV BLUEs of LL was negatively correlated with SD [*r* =  − 0.34, *P* =  < 0.0001] (Fig. 2). Similarly, significant negative correlations were also observed between combENV data set of LW and SD (*r* =  − 0.44, *P* =  < 0.0001), LW and PM_14D (*r* =  − 0.45, *P* =  < 0.0001), LW and PM_21D (*r* =  − 0.44, *P* =  < 0.0001) (Fig. 2, Supplementary Table S3). The regression analyses showed that the stem LL and LW were also significantly and negatively associated with SD (*r* =  − 0.34, *R*^2^ = 0.11, *P* = 1.7 × 10^−6^ for LL; *r* =  − 0.44, *R*^2^ = 0.19, *P* = 1.5 × 10^−10^ for LW) (Fig. 3b, Supplementary Fig. S2). However, stem LL and IL had significant positive correlation (*r* = 0.49, *P* =  < 0.0001), and similarly a significant positive correlation was also found to be associated between stem LW and IL (*r* = 0.42, *P* =  < 0.0001), LW and PM_14D (*r* = 0.40, *P* =  < 0.0001), LW and PM_21D (*r* = 0.47, *P* =  < 0.0001) (Fig. 2). The regression analyses between stem LL, stem LW with IL also showed positive correlation (*r* = 0.49, *R*^2^ = 0.24, *P* = 6.9 × 10^−13^ for LL, and *r* = 0.42, *R*^2^ = 0.17, *P* = 1.9 × 10^−9^ for LW) (Fig. 3a, Supplementary Fig. S2). Regression analyses among the stem LW, PM_14D, PM_21D with IL, and SD were presented in the Supplementary Fig. S2. Interestingly, a highly significant positive correlation was observed among stem LL, LW, PM_14D, and PM_21D for stem resistance across all the studied environments and combENV analyses (Fig. 2). The correlation between stem LL and LW was strong and positive in CARR_19 (*r* = 0.91), LANG_19 (*r* = 0.90, *P* =  < 0.0001), CARR_20 (*r* = 0.91, *P* =  < 0.0001), OSN_20 (*r* = 0.90, *P* =  < 0.0001), and combined (*r* = 0.94, *P* =  < 0.0001) environments. Highly significant correlations were also reported between stem LL and PM_14D (*r* = 0.83, *P* =  < 0.0001), LL and PM_21D (*r* = 0.75, *P* =  < 0.0001) (Fig. 2). These results suggest that stem LW, PM_14D, and PM_21D could serve as proxies for LL during assessment of stem resistance to *S. sclerotiorum* in rapeseed/canola. Therefore, breeders could select any of the phenotypic trait out of four to evaluate the resistance performance of the genotypes in response to *S. sclerotiorum* attack, which might need further verification.

### Correlation between days to flowering and sclerotinia stem rot resistance

The DF was significantly and negatively associated with combENV BLUEs of LL, LW, PM_14D, PM_21D (*r* =  − 0.39, *P* =  < 0.0001 for LL; *r* =  − 0.44, *P* =  < 0.0001 for LW; *r* =  − 0.49, *P* =  < 0.0001 for PM_14D; and *r* =  − 0.59, *P* =  < 0.0001 for PM_21D) (Fig. 2, Supplementary Table S3). The regression analyses showed that DF were negatively and significantly associated with stem LL, LW, PM_14D, and PM_21D (*r* =  − 0.39, *R*^2^ = 0.14, *P* = 4.0 × 10^−8^ for LL; *r* =  − 0.44, *R*^2^ = 0.19, *P* = 1.7 × 10^−10^ for LW; *r* =  − 0.49, *R*^2^ = 0.24, *P* = 5.9 × 10^−13^ for PM_14D; *r* =  − 0.59, *R*^2^ = 0.34, *P* = 2.2 × 10^−16^ for PM_21D) (Fig. 3c, Supplementary Fig. S2). These negative correlation results further confirmed that there is a connection between the DF and SSR resistance in *B. napus*, indicating that early flowering genotypes tend to be more vulnerable to the *S. sclerotiorum* attack with increased stem LL, LW, and plant mortality than the late maturing genotypes.

### Genotypic data and principal component analysis

**Figure 4:**
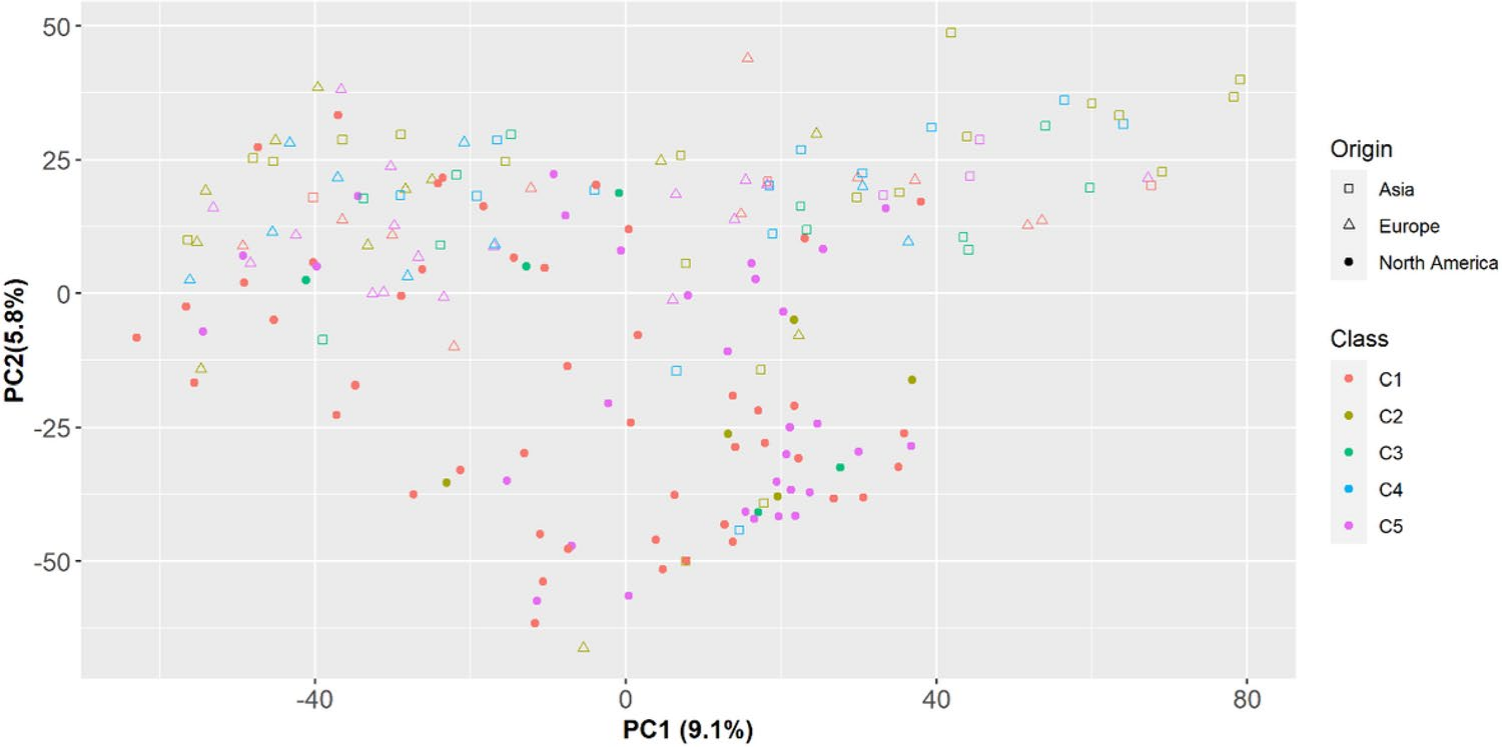
Population structure of rapeseed/canola genotypes as reflected by the scatter plot of PC1 and PC2 derived from a principal component analysis.

After eliminating markers with missing data greater than 25%, a total of 25,809 polymorphic SNPs with minor allele frequency (MAF) greater than 5% were obtained and employed for association analysis. The highest proportions of SNPs had MAF between 0.10 and 0.15 (20%) and between 0.05 and 0.10 (20%) (Supplementary Fig. S3). The other seven MAF classes represent between 3 and 14% each of the total markers. To scan the population stratification of the association panel, principal component analysis and kinship matrix were performed on the genotypes based on 25,809 SNPs. The first and second PCA accounted for 9.1 and 5.8% of the variance, respectively. The first 4 PCA accounted for 22% of the variance, and at PC4 the inflection point occurred, so we used four PCs in association mapping to avoid the confounding effect due to population structure. The model-based cluster analysis using the first four PCs suggested that there were 5 subgroups within the genotypes (Fig. 4).

### Marker-trait-association (MTA) analysis

**Figure 5:**
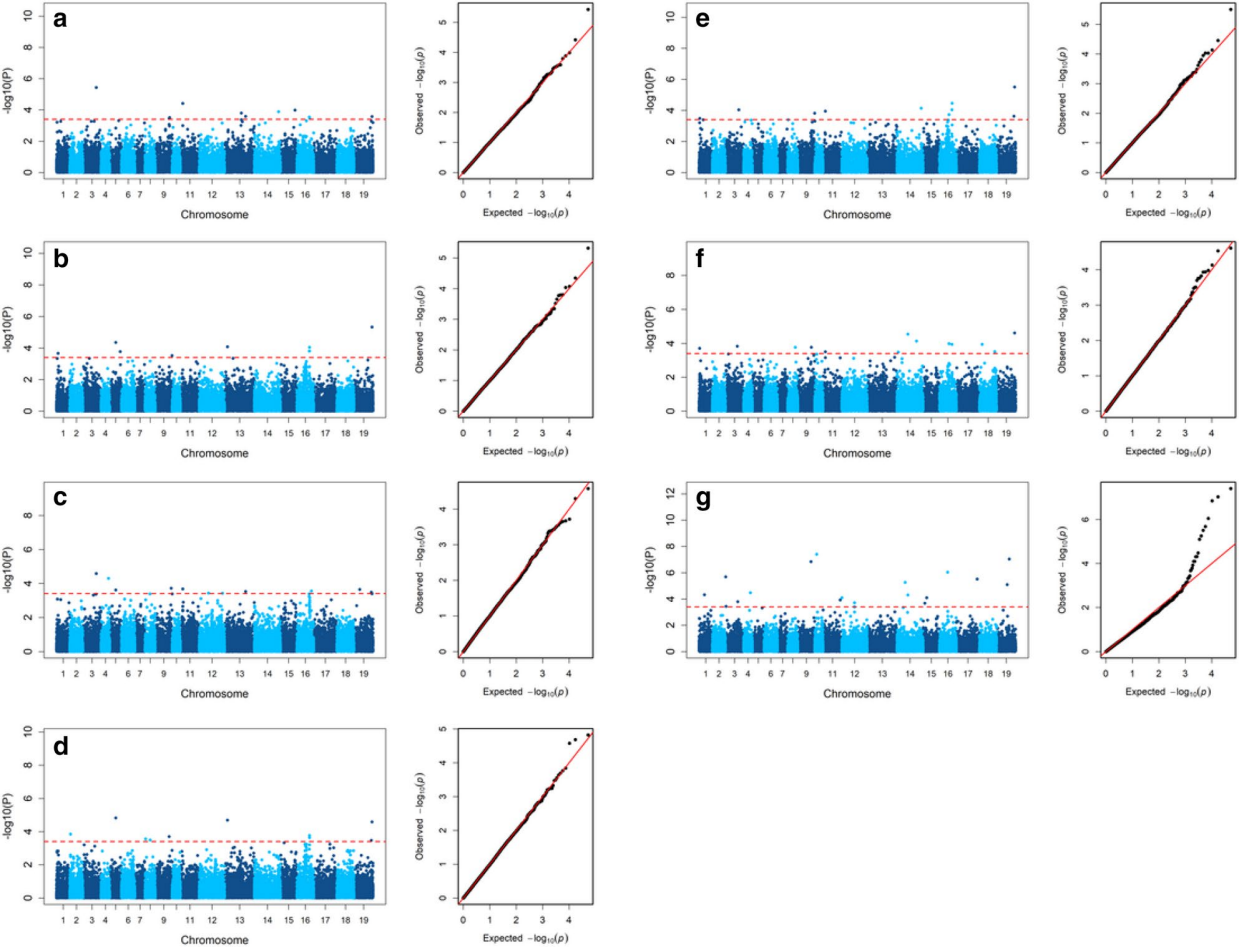
Manhattan and Q–Q plots showing the results of marker-trait association for sclerotinia stem rot resistance in 187 rapeseed/canola genotypes by the fixed and random model circulating probability unification (FarmCPU) GWAS model. (**a**) Lesion length, Carrington 2019; (**b**) lesion length, Langdon, 2019; (**c**) lesion length, Carrington 2020; (**d**) lesion length, Osnabrock 2020; (**e**) lesion length, combined data (CombENV); (**f**) plant mortality_14D, combined data (CombENV); and (**g**) plant mortality_21D, combined data (CombENV). The − log_10_ (*P*) values from a genome-wide scan are plotted against positions on each of the 19 chromosomes. Discontinued horizontal lines indicate the genome-wide significance threshold.

Association analyses using the phenotypic data and SNP marker data were conducted for each phenotypic trait for stem resistance (LL, LW and PM) in each year and with the combENV BLUEs data to identify best MTAs. To reduce false positive or false negative associations i.e. the chance of committing Type I and Type II errors, three different GWAS algorithms i.e. FarmCPU, MLM, and GEMMA-MLM, were used to identify the true MTAs. The population structures using four PCA and familial relatedness with kinship matrix were incorporated in the implemented MLM and GEMMA-MLM models to control pseudo associations. Incorporation of PCA and kinship matrix in the MLM models as covariates adjusts the correction tests to control false positives, but could not solve the confounding problem between the covariates and test marker, resulting false negatives^57^. FarmCPU is the model that effectively corrects both false positives and false negatives. In FarmCPU, the Multiple Loci Linear Mixed Model (MLMM) is divided into two parts: fixed effect model (FEM) and a random effect model (REM) and uses them iteratively. The first part (FEM) contains testing maker, one at a time, and multiple associated markers fitted as covariates to control false positives. To avoid the over fitting model problem in FEM, the associated markers are estimated through maximum likelihood method in REM by using them to define kinship^57^. In the current study, the SNPs detected in any two GWAS models were considered reliable and declared as significant SNP for the studied trait. Significant MTAs were determined on the basis of modified Bonferroni correction by calculating the effective number of independent tests (loci) from the tested 25,809 SNPs by Li and Ji^58^. The Q–Q plots generated from all GWAS analyses models of the analyzed phenotypic traits showed a sharp deviation from the expected *P* value distribution in the tail area, indicating that population structure and familial relatedness were well controlled and false positive associations were reduced (Fig. 5a–g).

### Stem lesion length

Association analysis was performed using BLUEs of LL of all four environments and combENV BLUEs separately (Fig. 5a–e, Supplementary Fig. S4). A total of 64 significant SNPs corresponding to 62 loci were identified at the level of [− log_10_ (*P*) ≥ 3.4; *P* ≤ 0.0004] by at least two of the GWAS models, and thus were regarded as more reliable. These SNPs were unevenly distributed among the *B. napus* chromosomes (Fig. 5a–e, Supplementary Fig. S4, Supplementary Table S4, 7). The majority of significant SNPs were detected on chromosomes A01 (5), A03 (5), A09 (6), C03 (8), and C06 (12). Significant SNPs that were present in the LD block on the same chromosome were regarded as single locus. Among these, thirty-eight significant SNPs were detected in two or more environments and in at least two of the GWAS tested models and explained phenotypic variance of the SNPs ranged from 4.5 to 9.9%. Allelic effects of these identified SNPs varied from − 0.84 to 0.83 (Supplementary Table S4, 7).

### Stem lesion width

GWAS analyses using LW BLUEs of all environments and combENV detected a total of 70 significant SNPs in 66 loci in at least one of the four environments and combENV datasets (Supplementary Fig. S5, Supplementary Table S5, 7). Out of these 70 significant SNPs, a total of 30 were found in at least two or more environments of two GWAS models out of three GWAS models. The estimated allelic effects of those 70 significant SNPs varied from − 6.83 to 7.55. The phenotypic variation accounted for by these SNP markers varied between 4.9 and 12.1% (Supplementary Table S5, 7). Manhattan and Q–Q plots summarizing the analysis of stem LW for SSR resistance by FarmCPU, MLM, and GEMMA-MLM are shown in (Supplementary Fig. S5).

### Plant mortality

CombENV BLUEs value of 14 and 21 dpi plant mortality were used to perform the GWAS analysis. Marker-trait-association analyses identified a total of 21 and 30 significant markers for PM_14D and PM_21D, respectively, which were commonly identified in at least two of the GWAS analysis models (Fig. 5f,g, Supplementary Fig. S6, Supplementary Table S6, 7). A total of 11 significant SNP markers were commonly found both in PM_14D and PM_21D (Supplementary Table S6). About 3.6 to 7.7% of the phenotypic variation were explained by these significant SNP markers. The estimated allelic effects were ranged between − 11.29 and 9.37 (Supplementary Table S6, 7). The MTAs resulting from FarmCPU, MLM, GEMMA-MLM for PM_14D_BLUP and PM_21D_BLUP were presented in the Manhattan and Q–Q plots (Supplementary Fig. S6).

### Candidate genes

The significant SNPs those were detected in at least two environments (four environments and combENV) were used to search for the candidate genes for sclerotinia stem rot resistance using “*ZS11*” reference genome sequence^47^. A total of 69 candidate genes with known functions associated with plant disease resistance mechanisms were identified within ± 50 kb of the respective significant SNPs. A list of these genes, their biological functions based on TAIR 10, Uniport-KB, annotations and corresponding details is provided (Supplementary Table S8). The candidate genes are involved in the biological process of defense response, defense response to fungus, programmed cell death, response to molecule of fungal origin, response to salicylic acid, indole glucosinolate biosynthetic process, induced systemic resistance, response to chitin, jasmonic acid mediated signaling pathway, ethylene-dependent systemic resistance, systemic acquired resistance, camalexin biosynthetic process, pattern recognition receptor signaling pathway, response to wounding, response to nematode, response to oxidative stress, toxin catabolic process, immune response, reactive oxygen species metabolic process, brassinosteroid mediated signaling pathway and other biological processes which might play key role in SSR resistance in rapeseed/canola (Supplementary Table S8).

### Genomic prediction

**Figure 6:**
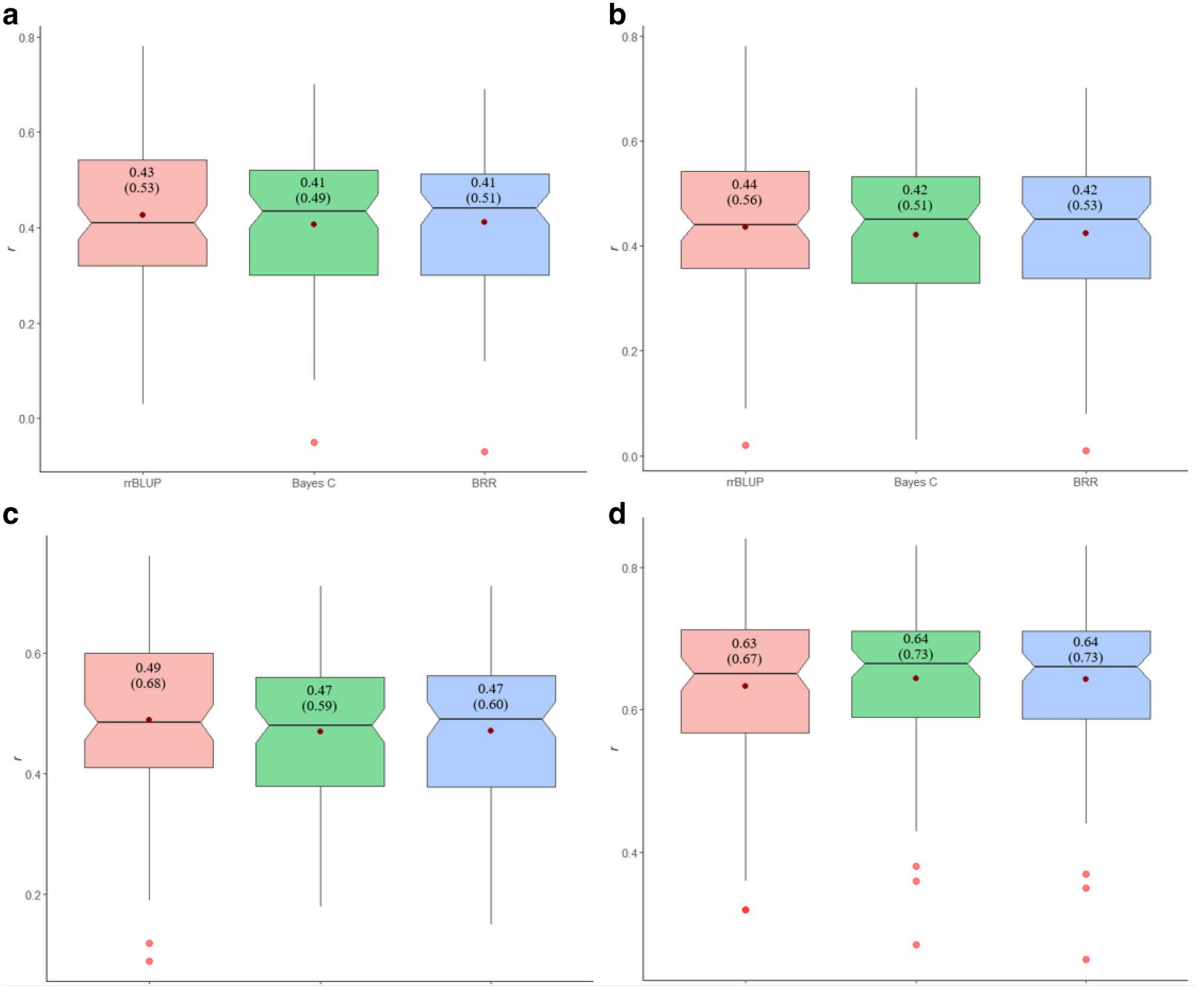
Predictive ability and accuracy for sclerotinia stem rot resistance associated phenotypic traits estimated from the fivefold cross-validation schemes of the association panel. (**a**) Boxplot showing the result of average predictive ability (*r*) (y axis) and accuracy for combENV stem lesion length, (**b**) combENV stem lesion width, (**c**) combENV plant mortality at 14 days post inoculation (PM_14D), (**d**) combENV plant mortality at 21 days post inoculation (PM_21D) with rrBLUP, Bayes C and Bayesisan ridge regression (BRR) models (x-axis). The boxes show second and third quartiles and wishkers show interquertile range. The red dot in each box plot represent the mean predictive ability. The number above horizontal black bars are the predictive ability (*r*) (at the top) and accuracy (below in brackets).

The three GS models used in this study showed more or less similar results across all traits that we evaluated (Fig. 6a–d, Supplementary Fig. S7). However, rrBLUP tended to generate better results than others in most cases. Slightly differences in the predictive abilities were observed among the used models for the studied traits. The predictive abilities applying genome-wide markers for stem LL, and LW traits varied from 0.06–0.51 and 0.12–0.52, respectively, for four individual environments (Supplementary Fig. S7). The LANG_19 environment showed the highest predictive ability for both LL (0.51) and LW (0.52) by rrBLUP, whereas the lowest predictive ability was observed 0.06 and 0.12 for the CARR_20 environment by Bayes C model (Supplementary Fig. S7). For environment-wise LL and LW traits, 1 to 9-unit and 0 to 6-unit differences in predictive ability was observed among the models, respectively, and the highest differences were found in CARR_20 environment for both traits. However, slightly differences (1 to 2-unit) in the predictive ability was observed for the combENV datasets of all traits. The average correlation between the GEBVs and the observed resistance to SSR by GP models were ranged by 0.41–0.43, 0.42–0.44, 0.47–0.49, and 0.63–0.64 for combENV LL, LW, PM_14D, and PM_21D, respectively (Fig. 6a–d). Overall, Bayes C and BRR models perform slightly poor over the rrBLUP model for all traits with an exception for PM_21D trait, where both Bayesian models resulted 1 unit increase in predictive ability than rrBLUP model. The predictive ability of PM_21D trait was about 47–56%, 43–52%, and 28–31% higher than the LL, LW, and PM_14D traits, respectively.

Since, the true breeding value of these traits are unknown, we estimate the approximate prediction accuracy to divide the correlation between the phenotypes and the mean predictive ability obtained through cross-validation sets by the square root of heritability ($\surd$*h*^2^). Therefore, we estimated the narrow-sense heritability based on the whole datasets using the same GS model used to fit the cross-validation sets. The genomic heritability varied, depending on the traits and used GS models as shown in Table 2. Thus, after using the genomic heritability, the estimated prediction accuracy ranged from about 0.49 to 0.53 for LL, 0.51 to 0.56 for LW, 0.59 to 0.68 for PM_14D, and 0.67 to 0.77 for PM_21D traits, depending on the used GS models (Fig. 6a–d). Overall, the predictive ability and accuracy results suggested that genomic predictions were stronger when plant mortality data were used rather than the lesion length and width.

**Table 2:** Genomic heritability (narrow-sense heritability) of the combined analyzed phenotypic traits of *B. napus* genotypes for sclerotinia stem rot obtained using all SNPs.

Model	Traits^a^	Narrow-sense heritability
rrBLUP	LL	0.65
Bayes C	LL	0.68
BRR	LL	0.65
rrBLUP	LW	0.6
Bayes C	LW	0.67
BRR	LW	0.64
rrBLUP	PM_14D	0.51
Bayes C	PM_14D	0.62
BRR	PM_14D	0.62
rrBLUP	PM_21D	0.89
Bayes C	PM_21D	0.77
BRR	PM_21D	0.77

^a^Traits: *LL* lesion length, *LW* lesion width, *PM_14D* plant mortality at 14 days post inoculation, *PM_21D* plant mortality at 21 days post inoculation.

## Discussion

Sclerotinia stem rot (SSR) is one of the most economically important and devastating fungal disease of rapeseed/canola that significantly limits seed yield, oil content, and oil quality worldwide. SSR is a highly heritable complex trait, controlled by many genes with minor additive effects^17–20^. Since, source of completely durable genetically resistant genotypes against this disease have not been identified to date in rapeseed/canola, breeding for SSR resistance is primarily dependent to a large extent on the utilization of partially resistant source^15,16^. In this study, we explored our rapeseed/canola diversity panel, including released cultivars, advanced breeding lines, and landraces from the different geographical regions with high genetic diversity against SSR in field trials conducted in four environments by inoculating plants artificially.

This study was designed to obtain the most accurate phenotypic and genotypic data possible. *S. sclerotiorum* isolates can vary widely in their virulence, or ability to cause damage to plants and consequently, plant genotypes may respond differently to different isolates^64,65^. We used a highly virulent *S. sclerotiorum* isolate, WM031, for this study. This selection allowed us to increase the infection efficiency and effective phenotypic screening for SSR resistance. The effective screening would allow us to the proper evaluation of the corresponding genotypes to produce reliable phenotypic value, which would ultimately improve the GWAS analyses to identify MTAs associated with SSR resistance. To generate stable phenotypic values, we have conducted the study at four location-years with three replications in each location. To mimic the natural sclerotinia infections on rapeseed/canola plants, we used agar-plug stem inoculation method to challenge the plants. This screening method has been implemented successfully by other researchers for the effective identification of SSR resistance^20,24,66^. Plants were inoculated during the flowering, because this is the most prevalent stage for SSR infection in natural field conditions^22^. The occurrence of SSR at the adult plant stage is a major cause for the seed yield loss and reduced oil content and quality. Therefore, we decided to identify the SSR resistance genotype source and resistance significant MTAs at the mature plant stage in order to incorporate the resistance into the elite canola breeding cultivars and to facilitate the MAS in rapeseed/canola breeding program. Hereafter, we analyzed 187 rapeseed/canola diverse genotypes for SSR resistance under field conditions. This germplasm primarily originates from North America, Europe, Asia and comprises of 26.7, 26.2, and 47.1%, respectively. Gyawali et al.^29^ carried out GWAS analyses for resistance to SSR under controlled conditions using 152 accessions collected from 18 countries consisting of 0.7% Australia, 69.7% Asia, 20.4% Europe, 7.2% North America, 0.7% South America and 1.3% from the unknown origin. Another GWAS study for SSR resistance conducted by Wu et al.^19^ used a panel of 448 germplasm accessions comprised of 93.8% (Asia), 1.1% (Australia), 1.1% (North America), and 4.0% (Europe) geographical origins. The geographic distribution of the genotypes used in our study provides a good coverage of world-wide germplasm accessions.

The reaction of the 187 rapeseed/canola diverse genotypes was evaluated and measured in three different ways, lesion length (LL), lesion width (LW), and plant mortality for SSR resistance. In our study, wider phenotypic variability was observed in the stem LL (2.3–9.0 cm), LW (19.3–81.4%), PM_14D (2.0–63%), and PM_21D (16–94%) against the disease infection, indicating that this diversity panel was ideal for performing GWAS. Phenotypic variability together with ideal diversity panel and high number of SNPs would potentially increase the effectiveness and efficiency of significant association detection via marker-trait-associations (MTAs)^67,68^. The lesion length on the main stem is the most commonly used phenotypic parameter in studies that evaluate the resistant performance of genotypes against SSR in rapeseed/canola^18–20,66^. The association study implemented by Gyawali et al.^29^ used lesion length and percentage of soft and collapsed lesions on the inoculated stem by noting depth of penetration for the assessment of SSR resistance. However, Wei et al.^18^, and Wu et al.^19^ used lesion length as a phenotypic trait for the evaluation of SSR resistance and GWAS analyses. In addition to LL, the LW and PM were also recorded. There were significant differences among the genotypes in relation to LL, LW, and PM at 14 dpi and 21 dpi. Results of this study clearly indicated that LW, PM_14D, PM_21D could be used as alternative phenotypic disease traits for the assessment of SSR resistance in rapeseed/canola. Since, plant mortality is directly related to yield performance, therefore it may be necessary to record plant mortality in addition to the other associated traits for sclerotinia phenotyping. Plant mortality has successfully been used by Shahoveisi et al.^24^. Li et al.^66^ evaluated 42 *B. napus* and 12 *B*. *juncea* genotypes for SSR resistance for PM at 21 dpi under field conditions and did not find any significant differences among the genotypes. Interestingly, we have found strong significant correlations among the LL, LW, PM_14D and PM_21D. Therefore, LW and PM could be used as an alternative phenotypic trait/parameter for breeders and pathologists to successfully differentiate and identify the potential SSR resistance genotypes under field conditions. To the best of our knowledge, this is the first to record the disease related LW phenotype to use as an alternative to LL and we demonstrated that there is a significantly high positive correlation between these two traits. The heritability of the stem resistance measured using LL was high which is consistent with previous studies^18–20^. Medium to high heritability for LW, PM_14D, and PM_21D was also estimated from the replicated multiple-location trials and combENV analyses, implying that phenotypic variation is mostly derived from genetic variance and phenotypic selection is effective for improving SSR resistance and subsequent association analyses to identify favorable alleles associated with SSR resistance to utilize in MAS.

Several agronomic traits such as plant height, canopy architecture, stem diameter, and flowering time were reported as associated with the sclerotinia disease severity in different crops such as canola, soybean, dry bean etc^20,66,69–72^. Therefore, the relationships between SSR disease phenotypic traits (LL, LW, PM_14D, PM_21D), and three agronomic traits such as FT, SD, and IL were also explored to assess whether they have direct or indirect effects on the SSR resistance. Results from this study showed that FT and SD had a significant and negative correlation with LL, LW, PM_14D, and PM_21D on *B. napus*. A similar association was reported in previous studies of this pathosystem^15,20,23,71,72^, and implied that early flowering genotypes were more prone to SSR susceptibility with increased stem LL, LW, and plant mortality. A similar association has been reported on other pathosystems, like *Arabidopsis-Verticillium dahlia*^73^; *Arabidopsis*-*Fusarium oxysporum*^74^; rice-*Pyricularia oryzae*^75^. Studies carried out by Wu et al.^71^ and Zhang et al.^72^ detected few co-localized QTL for the SSR resistance and FT in *B. napus*. They suggested a possible genetic linkage between these two traits. However, the underpinning genetic and molecular mechanisms controlling these associations are still not evident^71^. In addition to the effect of FT, Qasim et al.^20^ found a weak negative correlation between LL and SD, which is an agreement with our current study. Li et al.^66^ observed lower stem LL and plant mortality when the stem diameter was around 10 mm. However, increased lesion length and plant mortality were observed when SD was smaller or larger than 10 mm. Also, significant, positive, but moderate correlations were detected between IL and stem LL, IL and stem LW, IL and PM_14D, and IL and PM_21D. Therefore, this is an indication that evaluation of IL could be another useful parameter for an indirect selection for potential SSR disease resistant genotypes to use in the rapeseed/canola breeding program. Based on the findings from our study as well as from previous studies, effect of agronomic traits i.e. stem IL, and SD need to be taken into careful consideration for the breeders and pathologists to identify and select the accurate promising SSR resistance genotypes phenotypically from the field screening.

Bi-parental linkage mapping and association mappings have been used to dissect the complex traits such as SSR resistance in *B. napus* in order to identify the genetic loci conferring resistance. GWAS is a powerful genetic mapping strategy for the dissection of complex traits in plants^18,38,76^. Therefore, in this study, GWAS was implemented using three different models for SSR resistance associated traits i.e. LL, LW, PM_14D, and PM_21D with the objectives to maximizing opportunities to identify reliable and stable specific genomic regions and SNPs conferring SSR resistance in rapeseed/canola germplasm. This is the first time to use different single locus (MLM and GEMMA-MLM) and multi-locus (FarmCPU) GWAS models to identify common markers associated with this disease. The purpose of using three different GWAS softwares/algorithms was to reduce the chances of committing type I (false-positive association) and type II (false-negative association) errors. The identification of commonly detected SNPs simultaneously with multiple GWAS models and traits would improve the reliability of the detected MTAs associated with SSR resistance. As the SSR resistance is a quantitatively inherited complex trait and the number of SNP markers is larger than the sample size, it would be necessary to simultaneously use multiple methods for GWAS to identify stable MTAs. Bonferroni-Holm correction^77^ for multiple testing (*α* = 0.05) was too conservative, since it assumes that all the tests are independent but in reality, some SNPs may not be independent and they might be in linkage disequilibrium (LD) due to their physical distance or other associated factors. Moreover, with this Bonferroni-type correction, no significant associations between markers and evaluated traits in most of the environments were detected. The use of stringent significant probability threshold reduces the risk of accepting false positives but does not necessarily reduce the risk of rejecting true MTAs. Therefore, the significant threshold value for the association between SNP and traits were estimated by the method proposed by Li and Ji^58^. In this study, most of the significant SNP markers associated with the SSR resistance traits (LL, LW, PM_14D, PM_21D) detected in the environment-wise and combENV datasets showed small effects, explaining 3.6–12.1% of the observed phenotypic variance. These finding are in an agreement with previous genetic mapping studies (QTL and GWAS) on sclerotinia resistance. This suggests and validates that SSR resistance in *B. napus* is a complex genetic trait, quantitatively inherited and determined by multiple minor QTL with small effects^15,16,19–23,71^. Li et al.^66^ conducted an integrated and comparative QTL analyses for SSR resistance using previously identified QTLs from various mapping studies with the Darmor-bzh reference genome^47^ and determined that chromosomes A9 (22.5–27.5 Mb) and C6 (29.5–36.1 Mb) are conserved QTL regions. The putative disease resistance nucleotide-binding-site, leucine-rich-repeat (NBS-LRR) genes were found in this region in a cluster. GWAS analyses from our study revealed a total of 14 significant SNPs located on C6 (22.3–39.4 Mb) genomic regions, and 5 of them were located on chromosome C06 (33.2–34.1 Mb) region that was reported by Li et al.^26^. However, the SNP markers on chromosome C06 identified in this study have shown overlapping confidence intervals with the QTLs for stem resistance detected by Zhao et al.^15^, Wu et al.^22^, Wei et al.^18^, Wu et al.^19^, and Qasim et al.^20^. Identification of the SNPs in our study which aligned with previously identified overlapping genomic regions provide strong evidence that fine-mapping using large segregating mapping population could help us to narrow down the genomic regions. This may lead us to identify the putative candidate gene conferring SSR resistance in rapeseed/canola, and therefore, guide us for the map-based cloning of the sclerotinia resistance gene in future to assist MAS.

Several QTLs reported here were localized in the vicinity of QTL identified by other researchers. GWAS study conducted by Wu et al.^19^ identified five significant SNPs on chromosome A08 (15.09–15.10 Mb) region based on the alignment of Darmor-bzh^25^ reference genome. We detected six SNPs on chromosome A08 (13.7–22.9 Mb) region based on ‘ZS11’ reference genome sequence^47^. The ‘ZS11’ reference genome sequence was aligned with ‘Darmor-bzh’ reference genome^25^. Therefore, our finding was a close agreement with Wu et al.^19^. We also identified significant SNPs on chromosome C08 (SCM002776.2_29886188, SCM002776.2_37107013) which located near or overlapped with the identified QTL genomic regions of chromosome C8 (31.4–33.5 Mb, 38.2–38.5 Mb) reported by Wu et al.^22^. A significant SNP (SCM002777.2_46851981) for LL located on chromosome C09 repeatedly detected on multiple environments (CARR_19, CARR_20, OSN_20, CombENV) were found to be overlapped with the physical interval of *Sll19* for stem lesion length using petiole inoculation technique by Zhao et al.^15^. Moreover, another stable SNP marker (SCM002777.2_48885679) on chromosome C09 identified in almost all the environments and combENV with all the traits except PM_21D located at the physical position of 48.9 Mb in the ‘ZS11’ reference genome sequence. Detection of these stable genomic regions (46.8–48.9) on chromosome C09 in our current study as well as from the previous study provide an exciting opportunity to further explore these regions to develop molecular markers for future MAS in the rapeseed/canola breeding program for SSR resistance. Shahoveisi et al.^24^ reported QTL SR54.C3.1 associated with SSR resistance in the physical region of (23.4–31.6 Mb) on chromosome C03, the two SNPs [SCM002771.2_22853068 (22.9 Mb), SCM002771.2_27877818 (27.9 Mb)] in our study was found in close proximity or within the genomic regions. Moreover, we collected information on the previously identified QTLs, SNPs and their physical positions based on marker information from the past studies and compared it with our findings. In addition to the identification of significant SNPs in the previously detected genomic regions, to best of our knowledge, hereafter we are reporting new genomic regions on chromosome A09 (35.6–45.8 Mb) consisting of ten significant SNP markers, chromosome A03 (28.2–36.2 Mb) with seven significant SNPs, and chromosome A05 (15.9–28.6 Mb) with six markers are associated with SSR resistance.

One of the key objectives of GWAS is the identification and utilization of the candidate genes. Thereby we searched for candidate genes associated with disease resistance mechanisms that were located within 50 kb upstream and downstream of significant SNPs detected in at least two environments. We choose 50 kb because LD for this population is low (< 45 kb genome wise, < 21 kb for A genome and < 93 kb for C genome^78^. Based on these criteria, sixty-nine genes associated with defense response mechanisms were identified. A TIR-NB-LRR gene (LOC106415792) that putatively encodes *RPP1* proteins was located in the vicinity of SNP (SCM002777.2_48885679) detected in all the environments and combENV with stem LL, LW, PM_14D traits on chromosome C09. TIR-NB-LRR genes provide defense response against fungi through the activation of the salicylic acid (SA)-dependent resistance pathway^79^. SA has been known to be involved in the activation of defense response against biotrophic and hemi-biotrophic pathogens. However, recent findings suggest *S. sclerotiorum* has a brief biotrophic phase followed by a necrotrophic phase^80,81^. Our findings are in agreement with Nováková et al.^82^. Two annotated candidate genes, *WRKY transcription factor 33* (*WRKY33*) and a peroxidase C3-like, were detected 7.1 kb upstream and 4.6 kb downstream of marker SCM002772.2_65359864 on chromosome C4. This marker was detected using stem LL and LW data sets in multiple environments. *WRKY33* is involved in defense response to fungus and the camalexin biosynthetic processes which was found to be involved in providing resistance against *S. sclerotiorum*^83^. Wang et al.^84^ demonstrated that overexpression of *BnWRKY33* markedly enhanced resistance to *S. sclerotiorum* in *B. napus*. Another candidate gene annotated peroxidase C3-like to be involved in defense response. The remaining sixty-six genes reported in this study also encode proteins involved in the disease resistance mechanisms according to TAIR 10 and Uniport-KB.

Identification of stable QTL/MTAs is a prerequisite for their use in a breeding program to facilitate the MAS. In this study, thirty-three significant MTAs were found to be co-localized or in close proximity with the earlier bi-parental and GWAS mapping studies reporting QTL/MTAs that could be exploited and integrated for SSR resistance into the breeding program (Supplementary Table S7). To our knowledge, this is the first study that used three different GWAS algorithms and four phenotypic traits (stem LL, LW, PM_14D, PM_21D) under field conditions to identify genomic regions associated with reaction to *S. sclerotiorum*. Further, this is the first time that comprehensive phenotypic evaluation of three physiological traits, days to flowering, stem diameter, and stem internode length, indicate these traits play an important indirect role for the selection of SSR resistance genotypes in the field. Out of one-hundred thirty-three significant SNPs, nineteen of them were detected in at least two environments by at least two GWAS models and two phenotypic traits. Detection of stable and common MTAs with multiple traits, implementing multiple GWAS models in multiple environments could provide more confidence and reliability on the reported MTAs, and new genomic regions for SSR resistance from our current study.

Genomic selection is an effective genomic approach for the improvement of complex traits in crops^32,35,39,85^. GP models with the environment-wise BLUEs of stem LL, and LW resulted 0.06–0.51 and 0.12–0.52 predictive abilities, respectively. Moreover, predictive abilities implementing three GS models i.e. rrBLUP, Bayes C and BRR for the combENV datasets for stem LL, LW, PM_14D, and PM_21D for SSR resistance were 0.41 to 0.3, 0.42–0.44, 0.47–0.49, and 0.63–0.64, respectively. These results clearly demonstrate that genome-wide markers are efficient in predicting SSR resistance. None of the models outperform than the others, with an exception for CARR_20 environment, consistent with results obtained by other researchers^41,86,87^. The observed differences in the predictive abilities among the used models for the combENV traits were mostly 1 to 2 units, which were likely due to GS model’s underlying assumptions. For example, rrBLUP model assumes that all the marker effects have identical variance and all markers effects have drawn from the same gaussian/normal distribution^33^. Bayes C assumes a priori that markers have normally distributed effects with probability π and no effect with probability (1 − π)^88^. BRR produces homogeneous shrinkage of all marker effects towards zero and yields a normal distribution of the marker effects^89^. The consistent results were also reported by Derbyshire et al.^41^,where Bayesian models perform similar or worse than G-BLUP model to predict *S. sclerotiorum* resistance in *B. napus*. The predictive ability in this study ranging from medium to high, were comparable or higher than the estimated predictive ability of SSR resistance in two previous studies^18,41^. The differences in predictive ability could be attributed due to the difference in populations, diversity, linkage disequilibrium, and trait heritability^32,90,91^. However, the estimated predictive ability was more or less similar with the reported predictive ability by Derbyshire et al.^41^ when lesion length data was used as a target trait. Interestingly, in our current study, using PM_21D data we achieved 47–56% increase in predictive ability. The results suggest that use of PM_21D data rather than pathogen spreading (stem lesion length, and lesion width) could be used as a useful phenotypic trait, which would potentially enable the breeders to achieve higher predictive ability and leading towards the selection of superior genotypes for SSR resistance breeding in rapeseed/canola. GWAS results from this study indicated that most of the significant SNPs explained only 3.6–12.1% of the phenotypic variance. However, most of the identified significant SNPs or QTL from the GWAS and bi-parental linkage mapping studies also showed small effects, explaining less than 10% of the observed phenotypic variance^15,16,19,21,23^. Thus, genomic selection offers promising opportunities by capturing the effects of both minor and major genes to exploit the full genetic potential over selection based on few significant markers for the improvement of SSR resistance in rapeseed/canola. In future, this study could be further improved by including more genotypes, as well as integrating other independent biological information.

In this study, we have identified resistant genotypes from a genetically diverse resource will serve as a potential donor for improving canola cultivars with SSR resistance at North Dakota State University canola breeding program. The use of multiple phenotypic data sets and GWAS models used on data collected in multiple field environments allowed for the detection of one-hundred thirty-three significant SNPs, some of them were in novel regions of the genome. Some SNPs were detected in multiple datasets and models, suggesting their association with the resistant trait may be stronger than that of others. At the same time, they validate the notion that multiple approaches, e.g., phenotypic data sets and GWAS models, may yield additional information that otherwise would not be captured. The significant stable and new MTAs detected from this study could be used for future MAS of SSR resistance in rapeseed/canola breeding. Further, the strong and significant correlation detected among the phenotypic traits suggested that, stem LW, PM_14D and PM_21D could be used as proxies for stem LL when evaluating genotypes for their reaction to SSR. This study also assessed the potential of GP using different GS models and revealed a medium to high predictive ability depending on various phenotypic traits. Our results suggest that GS holds promise for the improvement of SSR resistance, and its application would enable the breeders for early SSR resistance genotype selection to accelerate the breeding efficiency by reducing the need to phenotype large number of genotypes in the field at maturing stage.

## Supplementary Information

Supplementary Information 1.

Supplementary Information 2.

Supplementary Information 3.

Supplementary Information 4.

Supplementary Information 5.

Supplementary Information 6.

Supplementary Information 7.

Supplementary Information 8.

Supplementary Information 9.
