# Supplementary material for: Genome-wide association mapping and genomic prediction for adult stage sclerotinia stem rot resistance in Brassica napus (L) under field environments
Source: Sci Rep. 2021 Nov 5;11:21773. doi: 10.1038/s41598-021-01272-9 (PMC8571315; doi:10.1038/s41598-021-01272-9)
Supplement: Supplementary file 2 — Supplementary Information 2. [file 41598_2021_1272_MOESM2_ESM.docx]

**Supplementary Table S2.** Descriptive statistics of sclerotinia stem rot disease traits.

**Table S2:** Environment wise analysis of variance (ANOVA) for stem lesion length (LL) in 189 canola/rapeseed genotypes

CARRINGTON_2019

| Source of variation | df | Mean square | F value | *P* value | *H^2^* |
| --- | --- | --- | --- | --- | --- |
| Replicates | 2 | 18.05 | 11.50^***^ | <.0001 | 0.70 |
| Genotypes | 188 | 10.17 | 3.32^***^ | <.0001 |  |
| Genotypes x Replicates /Exp. Error | 376 | 3.07 |  |  |  |

*** Differences were significant at *P* < 0.0001 levels of significance

LANGDON_2019

| Source of variation | df | Mean square | F value | *P* value | *H^2^* |
| --- | --- | --- | --- | --- | --- |
| Replicates | 2 | 185.17 | 82.53^***^ | <.0001 | 0.64 |
| Genotypes | 188 | 14.50 | 2.76^***^ | <.0001 |  |
| Genotypes x Replicates /Exp. Error | 376 | 5.25 |  |  |  |

*** Differences were significant at P < 0.0001 levels of significance

CARRINTON_2020

| Source of variation | df | Mean square | F value | *P* value | *H^2^* |
| --- | --- | --- | --- | --- | --- |
| Replicates | 2 | 11.96 | 9.38^***^ | <.0001 | 0.78 |
| Genotypes | 188 | 8.57 | 4.45^***^ | <.0001 |  |
| Genotypes x Replicates /Exp. Error | 376 | 1.93 |  |  |  |

*** Differences were significant at *P* < 0.0001 levels of significance

OSNABROCK_2020

| Source of variation | df | Mean square | F value | *P* value | *H^2^* |
| --- | --- | --- | --- | --- | --- |
| Replicates | 2 | 269.41 | 108.55^***^ | <.0001 | 0.70 |
| Genotypes | 188 | 17.97 | 3.35^***^ | <.0001 |  |
| Genotypes x Replicates /Exp. Error | 376 | 5.36 |  |  |  |

*** Differences were significant at *P* < 0.0001 levels of significance

**Table S2.** Analysis of variance for stem lesion length (LL) in 189 canola/rapeseed genotypes across the 4 environments

| Source of variation | df | Mean square | F value | *P* value | H^2^ |
| --- | --- | --- | --- | --- | --- |
| Environment | 3 | 718.317246 |  |  | 0.89 |
| Replicates (Environment) | 8 | 121.145885 |  |  |  |
| Genotypes | 188 | 37.498868 | 8.86^***^ | <.0001 |  |
| Genotypes x Environment | 564 | 4.230666 | 2.22^***^ | <.0001 |  |
| Experimental Error | 1504 | 1.90967 |  |  |  |

*** Differences were significant at *P* < 0.0001 levels of significance

**Table S2.** Environment wise Analysis of variance for stem lesion width (LW) in 189 canola/rapeseed genotypes

CARRINGTON_2019

| Source of variation | df | Mean square | F value | *P* value | *H^2^* |
| --- | --- | --- | --- | --- | --- |
| Replicates | 2 | 1584.72 | 11.29 | <.0001 | 0.65 |
| Genotypes | 188 | 785.36 | 2.89 | <.0001 |  |
| Genotypes x Replicates /Exp. Error | 376 | 271.38 |  |  |  |

*** Differences were significant at *P* < 0.0001 levels of significance

LANGDON_2019

| Source of variation | df | Mean square | F value | *P* value | *H^2^* |
| --- | --- | --- | --- | --- | --- |
| Replicates | 2 | 52245.34 | 207.67 | <.0001 | 0.56 |
| Genotypes | 188 | 1685.78 | 2.26 | <.0001 |  |
| Genotypes x Replicates /Exp. Error | 376 | 746.58 |  |  |  |

*** Differences were significant at *P* < 0.0001 levels of significance

CARRINTON_2020

| Source of variation | df | Mean square | F value | *P* value | *H^2^* |
| --- | --- | --- | --- | --- | --- |
| Replicates | 2 | 1842.70 | 12.37 | <.0001 | 0.72 |
| Genotypes | 188 | 763.75 | 3.51 | <.0001 |  |
| Genotypes x Replicates /Exp. Error | 376 | 217.50 |  |  |  |

*** Differences were significant at *P* < 0.0001 levels of significance

OSNABROCK_2020

| Source of variation | df | Mean square | F value | *P* value | *H^2^* |
| --- | --- | --- | --- | --- | --- |
| Replicates | 2 | 14753.21 | 60.49 | <.0001 | 0.63 |
| Genotypes | 188 | 1457.76 | 2.72 | <.0001 |  |
| Genotypes x Replicates /Exp. Error | 376 | 536.39 |  |  |  |

*** Differences were significant at *P* < 0.0001 levels of significance

**Table S2.** Analysis of variance for stem lesion width (LW) in 189 canola/rapeseed genotypes across the 4 environments

| Source of variation | df | Mean square | F value | *P* value | *H^2^* |
| --- | --- | --- | --- | --- | --- |
| Environment | 3 | 54044.82 |  |  | 0.86 |
| Replicates (Environment) | 8 | 17606.49 |  |  |  |
| Genotypes | 188 | 3215.55 | 6.96 | <.0001 |  |
| Genotypes x Environment | 564 | 462.18 | 2.34 | <.0001 |  |
| Experimental Error | 1504 | 442.96 |  |  |  |

*** Differences were significant at *P* < 0.0001 levels of significance

**Table S2.** Environment wise Analysis of variance for stem diameter (SD) in 189 canola/rapeseed genotypes

CARRINGTON_2019

| Source of variation | df | Mean square | F value | *P* value | *H^2^* |
| --- | --- | --- | --- | --- | --- |
| Replicates | 2 | 58.58 | 12.24^***^ | <.0001 | 0.73 |
| Genotypes | 188 | 33.04 | 3.69^***^ | <.0001 |  |
| Genotypes x Replicates /Exp. Error | 376 | 8.95 |  |  |  |

*** Differences were significant at *P* < 0.0001 levels of significance

LANGDON_2019

| Source of variation | df | Mean square | F value | *P* value | *H^2^* |
| --- | --- | --- | --- | --- | --- |
| Replicates | 2 | 57.20 | 25.06^***^ | <.0001 | 0.65 |
| Genotypes | 188 | 17.77 | 2.88^***^ | <.0001 |  |
| Genotypes x Replicates /Exp. Error | 376 | 6.16 |  |  |  |

*** Differences were significant at *P* < 0.0001 levels of significance

CARRINGTON_2020

| Source of variation | df | Mean square | F value | *P* value | *H^2^* |
| --- | --- | --- | --- | --- | --- |
| Replicates | 2 | 70.55 | 26.15^***^ | <.0001 | 0.90 |
| Genotypes | 188 | 49.43 | 9.86^***^ | <.0001 |  |
| Genotypes x Replicates /Exp. Error | 376 | 5.01 |  |  |  |

*** Differences were significant at *P* < 0.0001 levels of significance

OSNABROCK_2020

| Source of variation | df | Mean square | F value | *P* value | *H^2^* |
| --- | --- | --- | --- | --- | --- |
| Replicates | 2 | 294.30 | 60.15^***^ | <.0001 | 0.73 |
| Genotypes | 188 | 31.41 | 3.64^***^ | <.0001 |  |
| Genotypes x Replicates /Exp. Error | 376 | 8.62 |  |  |  |

*** Differences were significant at *P* < 0.0001 levels of significance

**Table S2.** Analysis of variance for stem diameter (SD) in 189 canola/rapeseed genotypes across the 4 environments

| Source of variation | df | Mean square | F value | *P* value | *H^2^* |
| --- | --- | --- | --- | --- | --- |
| Environment | 3 | 327.29 |  |  | 0.88 |
| Replicates (Environment) | 8 | 120.16 |  |  |  |
| Genotypes | 188 | 96.86 | 8.25^***^ | <.0001 |  |
| Genotypes x Environment | 564 | 11.74 | 3.18^***^ | <.0001 |  |
| Experimental Error | 1504 | 3.69 |  |  |  |

*** Differences were significant at *P* < 0.0001 levels of significance

**Table S2.** Environment wise Analysis of variance for stem internode length (IL) in 189 canola/rapeseed genotypes

CARRINGTON_2019

| Source of variation | df | Mean square | F value | *P* value | *H^2^* |
| --- | --- | --- | --- | --- | --- |
| Replicates | 2 | 139.57 | 32.66^***^ | <.0001 | 0.67 |
| Genotypes | 188 | 31.03 | 3.05^***^ | <.0001 |  |
| Genotypes x Replicates /Exp. Error | 376 | 10.17 |  |  |  |

*** Differences were significant at *P* < 0.0001 levels of significance

LANGDON_2019

| Source of variation | df | Mean square | F value | *P* value | *H^2^* |
| --- | --- | --- | --- | --- | --- |
| Replicates | 2 | 213.75 | 40.43^***^ | <.0001 | 0.77 |
| Genotypes | 188 | 44.30 | 4.29^***^ | <.0001 |  |
| Genotypes x Replicates /Exp. Error | 376 | 10.32 |  |  |  |

*** Differences were significant at *P* < 0.0001 levels of significance

CARRINGTON_2020

| Source of variation | df | Mean square | F value | *P* value | *H^2^* |
| --- | --- | --- | --- | --- | --- |
| Replicates | 2 | 2.55 | 0.27^†^ | 0.7624 | 0.55 |
| Genotypes | 188 | 27.27 | 2.24^***^ | <.0001 |  |
| Genotypes x Replicates /Exp. Error | 376 | 12.20 |  |  |  |

† Differences were non-significant at *P* < 0.05 levels of significance

*** Differences were significant at *P* < 0.0001 levels of significance

OSNABROCK_2020

| Source of variation | df | Mean square | F value | *P* value | *H^2^* |
| --- | --- | --- | --- | --- | --- |
| Replicates | 2 | 53.53 | 5.06^*^ | 0.0064 | 0.75 |
| Genotypes | 188 | 61.87 | 3.99^***^ | <.0001 |  |
| Genotypes x Replicates /Exp. Error | 376 | 15.50 |  |  |  |

* Differences were significant at *P* < 0.01 levels of significance

*** Differences were significant at *P* < 0.0001 levels of significance

**Table S2.** Analysis of variance for stem internode length (IL) in 189 canola/rapeseed genotypes across the 4 environments

| Source of variation | df | Mean square | F value | *P* value | *H^2^* |
| --- | --- | --- | --- | --- | --- |
| Environment | 3 | 1993.77 |  |  | 0.79 |
| Replicates (Environment) | 8 | 102.35 |  |  |  |
| Genotypes | 188 | 99.66 | 4.86^***^ | <.0001 |  |
| Genotypes x Environment | 564 | 20.51 | 2.78^***^ | <.0001 |  |
| Experimental Error | 1504 | 7.37 |  |  |  |

*** Differences were significant at *P* < 0.0001 levels of significance

**Table S2.** Analysis of variance for plant mortality at 14 DAI (PM_14D) in 189 canola/rapeseed genotypes across the 4 environments

| Source of variation | df | Mean square | F value | *P* value | *H^2^* |
| --- | --- | --- | --- | --- | --- |
| Environment | 3 | 115220.31 |  |  | 0.90 |
| Replicates (Environment) | 8 | 4652.83 |  |  |  |
| Genotypes | 188 | 1696.34 | 5.63*** | <.0001 |  |
| Genotypes x Environment | 564 | 434.10 | 1.44*** | <.0001 |  |
| Experimental Error | 1504 | 301.45 |  |  |  |

*** Differences were significant at *P <* 0.0001 levels of significance

**Table S2.** Analysis of variance for plant mortality at 21 DAI (PM_21D) in 189 canola/rapeseed genotypes across the 4 environments

| Source of variation | df | Mean square | F value | *P* value | *H^2^* |
| --- | --- | --- | --- | --- | --- |
| Environment | 3 | 43802.68 |  |  | 0.96 |
| Replicates (Environment) | 8 | 4290.24 |  |  |  |
| Genotypes | 188 | 2036.98 | 6.69^***^ | <.0001 |  |
| Genotypes x Environment | 564 | 231.19 | 0.76^†^ | 0.9999 |  |
| Experimental Error | 1504 | 304.30 |  |  |  |

† Differences were non-significant at *P* < 0.05 levels of significance

*** Differences were significant at *P* < 0.0001 levels of significance

**Table S2.** Environment wise Analysis of variance for days to flowering (DF) in 189 canola/rapeseed genotypes

CARRINGTON_2019

| Source of variation | df | Mean square | F value | *P* value | *H^2^* |
| --- | --- | --- | --- | --- | --- |
| Replicates | 2 | 0.94 | 0.27^†^ | 0.7649 | 0.98 |
| Genotypes | 188 | 222.58 | 63.62^***^ | <.0001 |  |
| Genotypes x Replicates /Exp. Error | 376 | 3.50 |  |  |  |

† Differences were non-significant at *P* < 0.05 levels of significance

*** Differences were significant at *P* < 0.0001 levels of significance

LANGDON_2019

| Source of variation | df | Mean square | F value | *P* value | *H^2^* |
| --- | --- | --- | --- | --- | --- |
| Replicates | 2 | 8.05 | 2.33^†^ | 0.0989 | 0.99 |
| Genotypes | 188 | 232.35 | 67.14^***^ | <.0001 |  |
| Genotypes x Replicates /Exp. Error | 376 | 3.46 |  |  |  |

† Differences were non-significant at *P* < 0.05 levels of significance

*** Differences were significant at *P* < 0.0001 levels of significance

CARRINGTON_2020

| Source of variation | df | Mean square | F value | *P* value | *H^2^* |
| --- | --- | --- | --- | --- | --- |
| Replicates | 2 | 19.18 | 3.99^*^ | 0.0192 | 0.98 |
| Genotypes | 188 | 200.76 | 41.81^***^ | <.0001 |  |
| Genotypes x Replicates /Exp. Error | 376 | 4.80 |  |  |  |

* Differences were significant at *P* < 0.05 levels of significance

*** Differences were significant at *P* < 0.0001 levels of significance

OSNABROCK_2020

| Source of variation | df | Mean square | F value | *P* value | *H^2^* |
| --- | --- | --- | --- | --- | --- |
| Replicates | 2 | 22.94 | 1.89^†^ | 0.152 | 0.95 |
| Genotypes | 188 | 249.98 | 20.64^***^ | <.0001 |  |
| Genotypes x Replicates /Exp. Error | 376 | 12.11 |  |  |  |

† Differences were non-significant at *P* < 0.05 levels of significance

*** Differences were significant at *P* < 0.0001 levels of significance

**Table S2.** Analysis of variance for days to flowering (DF) in 189 canola/rapeseed genotypes across the 4 environments

| Source of variation | df | Mean square | F value | *P* value | *H^2^* |
| --- | --- | --- | --- | --- | --- |
| Environment | 3 | 12757.94 |  |  | 0.98 |
| Replicates (Environment) | 8 | 12.78 |  |  |  |
| Genotypes | 188 | 847.57 | 142.10^***^ | <.0001 |  |
| Genotypes x Environment | 564 | 19.37 | 3.25^***^ | <.0001 |  |
| Experimental Error | 1504 | 5.96 |  |  |  |

*** Differences were significant at *P* < 0.0001 levels of significance
