# Supplementary material for: Genome-wide association mapping and genomic prediction for adult stage sclerotinia stem rot resistance in Brassica napus (L) under field environments
Source: Sci Rep. 2021 Nov 5;11:21773. doi: 10.1038/s41598-021-01272-9 (PMC8571315; doi:10.1038/s41598-021-01272-9)
Supplement: Supplementary file 9 — Supplementary Information 9. [file 41598_2021_1272_MOESM9_ESM.pdf]

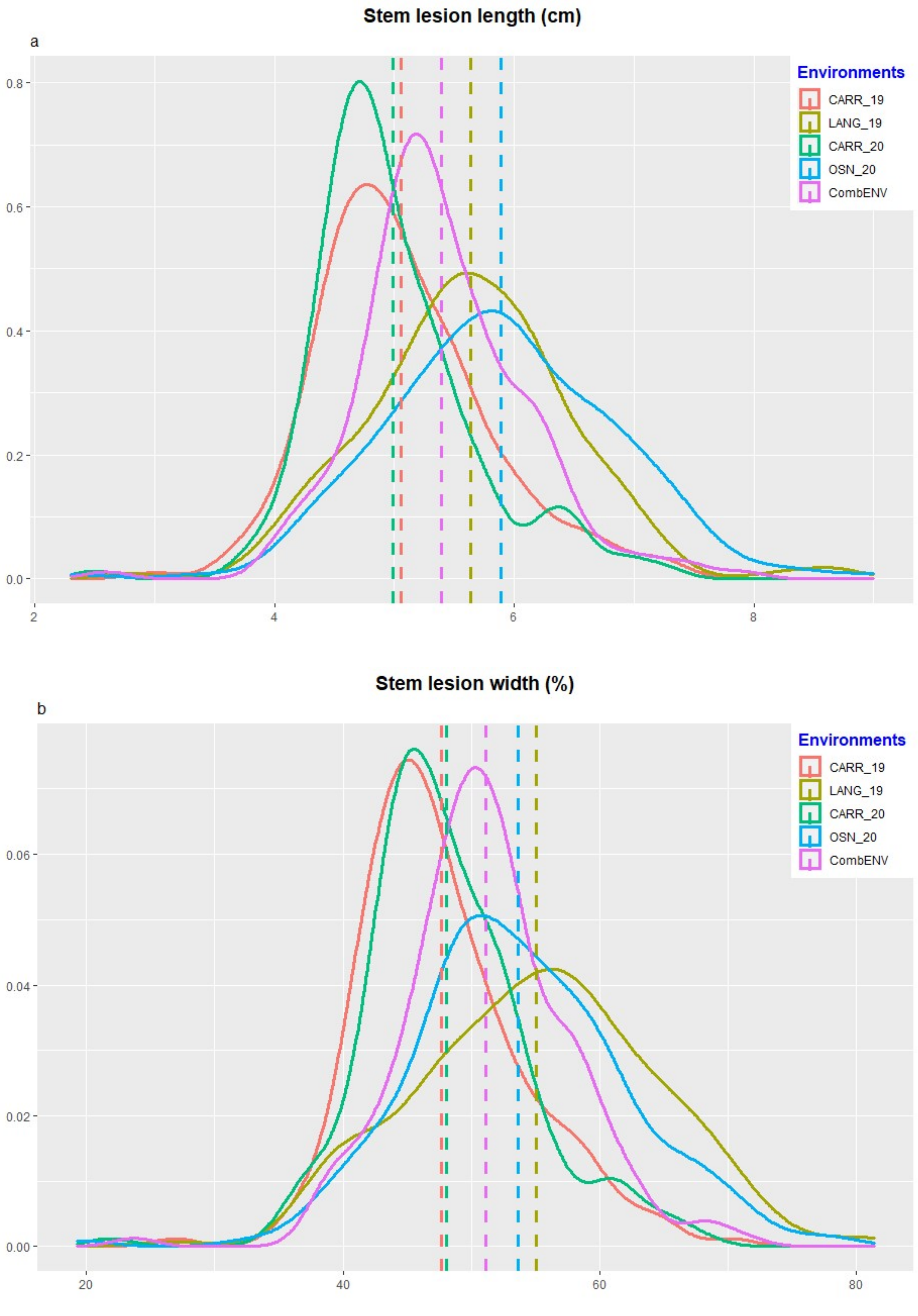
Stem lesion length (cm)
a
0.8
0.6
0.4
0.2
0.0
2
4
6
8
Environments
CARR_19
LANG_19
CARR_20
OSN_20
CombENV
Stem lesion width (%)
b
0.06
0.04
0.02
0.00
20
40
60
80
Environments
CARR_19
LANG_19
CARR_20
OSN_20
CombENV

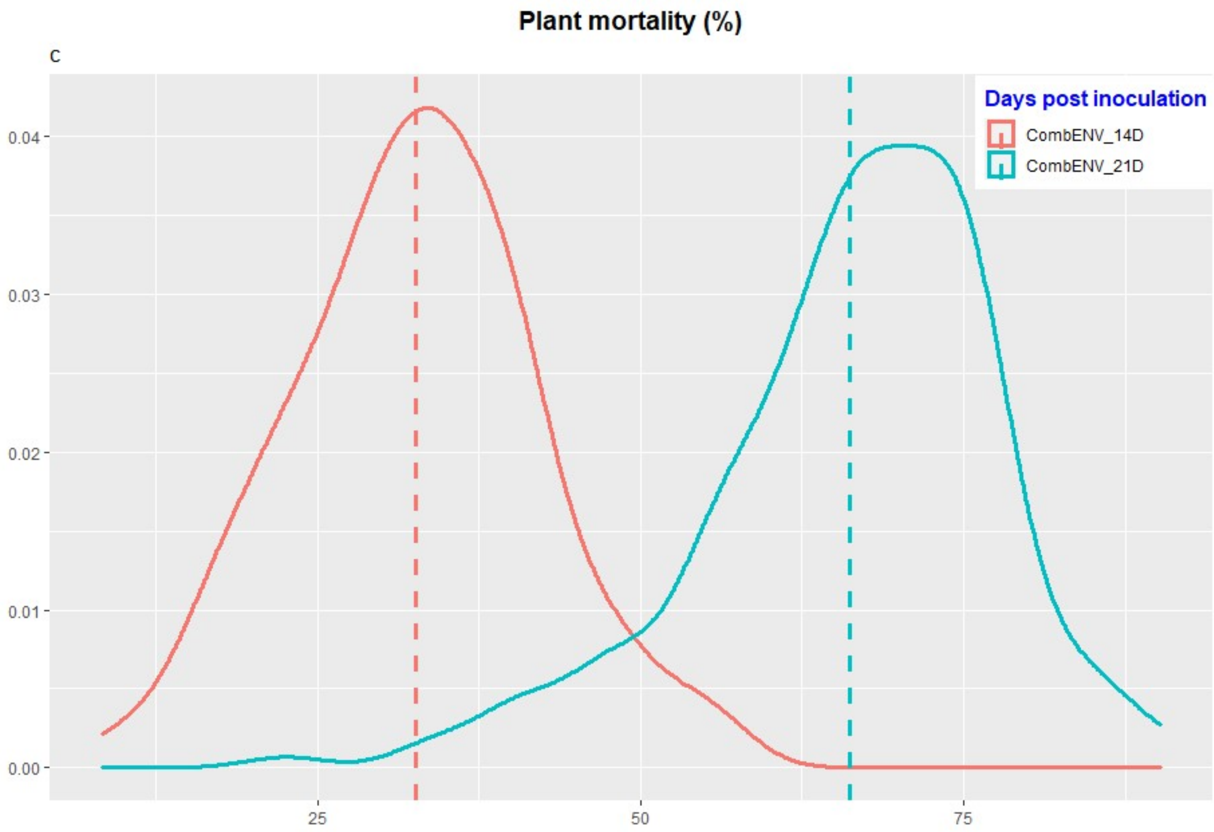

Plant mortality (%)
c
0.04
0.03
0.02
0.01
0.00
25
50
75
Days post inoculation
CombENV_14D
CombENV_21D

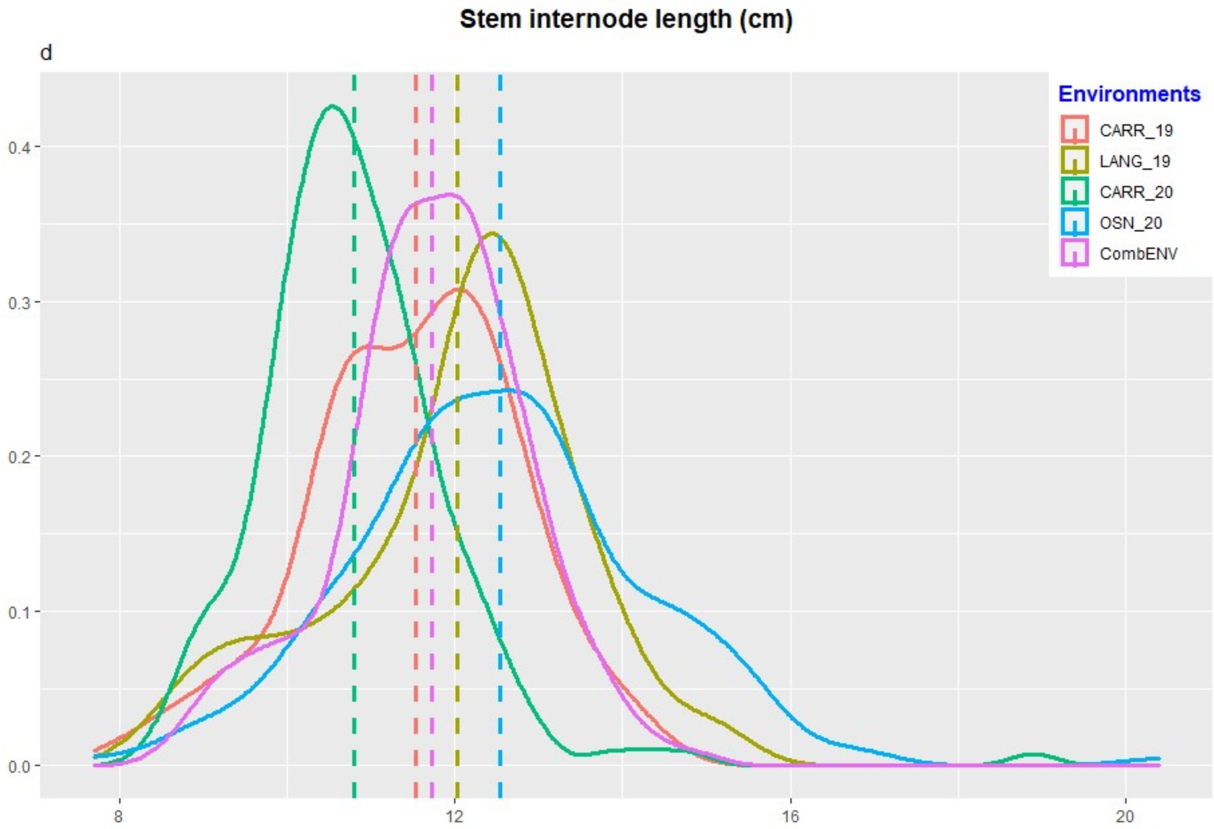

Stem internode length (cm)
d
0.4
0.3
0.2
0.1
0.0
8
12
16
20
Environments
CARR_19
LANG_19
CARR_20
OSN_20
CombENV

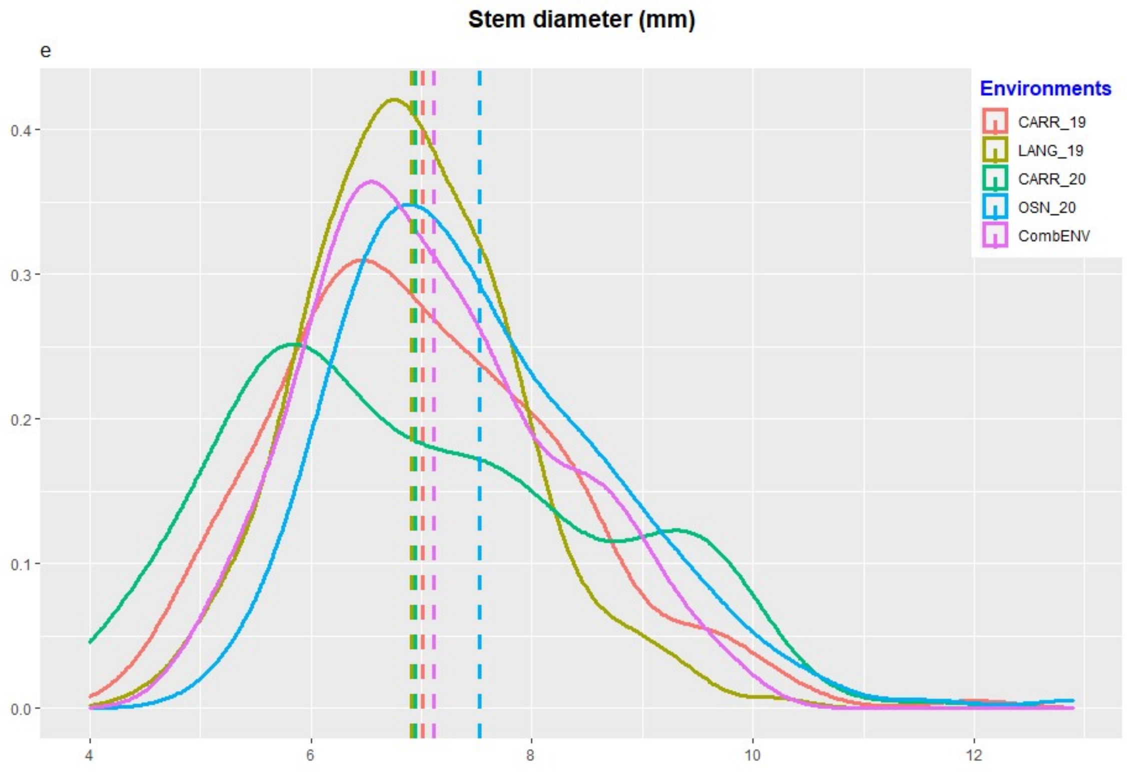

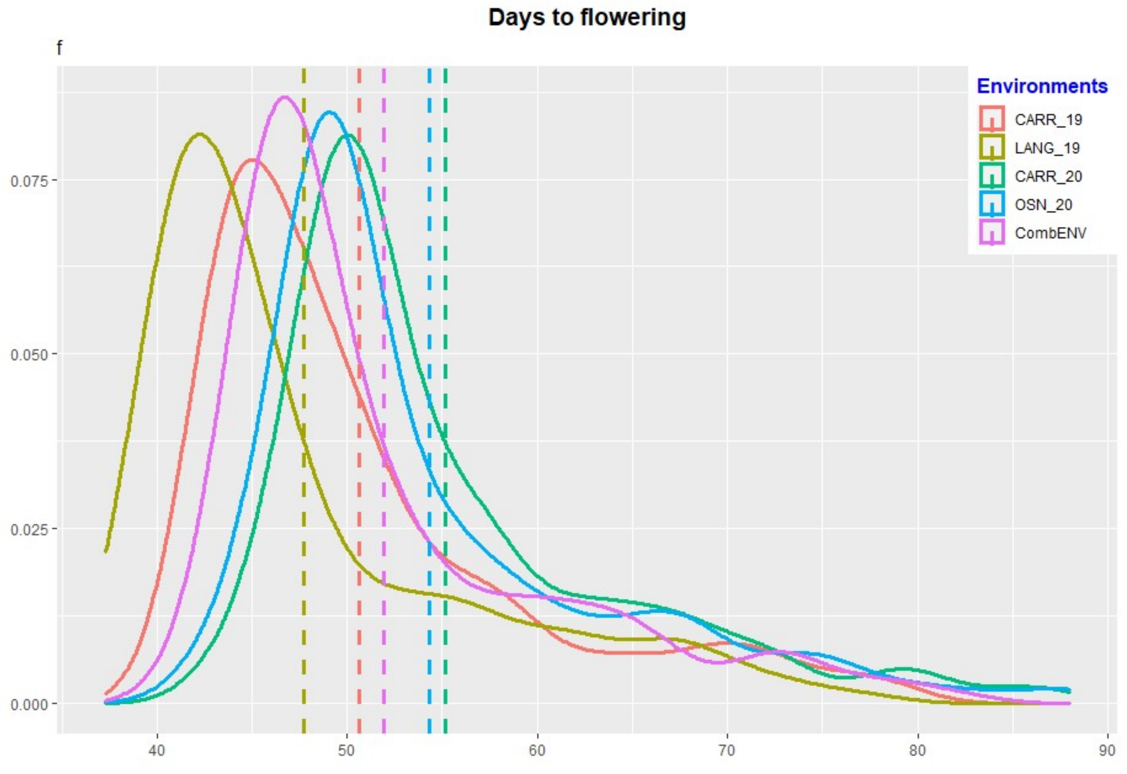

Supplementary Figure S1. Phenotypic distribution of different traits for sclerotinia stem rot disease a) stem lesion length, b) stem lesion width, c) plant mortality, d) stem internode length, e) stem diameter, and f) days to flowering in four environments, Carrington in 2019 (CARR_19), 2020 (CARR_20), Langdon in 2019 (LANG_19), Osnabrock in 2020 (OSN_20), and combined best linear unbiased estimates (CombENV_BLUEs) across all four environments. Mean values are represented by the vertical dashed line in the density distribution plot.

a

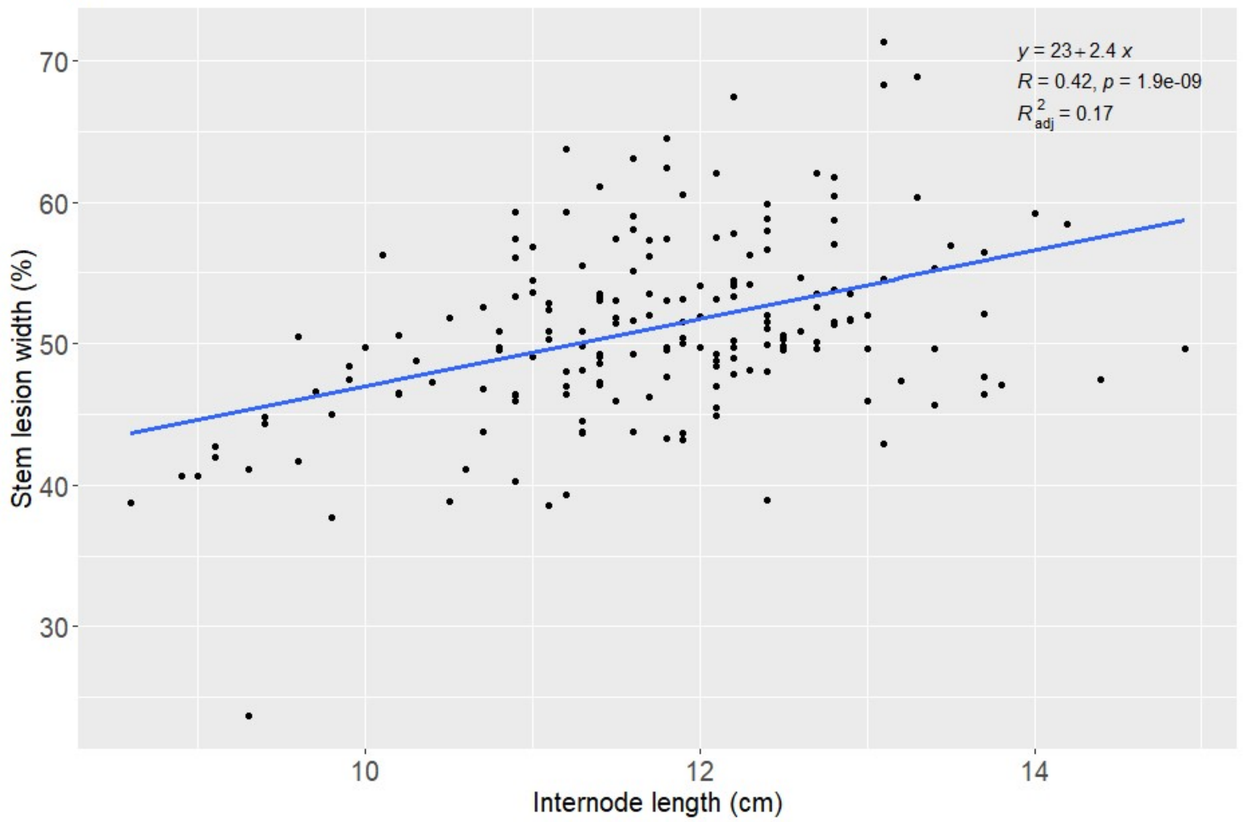

$y = 23 + 2.4\ x$
$R = 0.42,\ p = 1.9e\text{-}09$
$R^2_{adj} = 0.17$
Stem lesion width (%)
Internode length (cm)

b

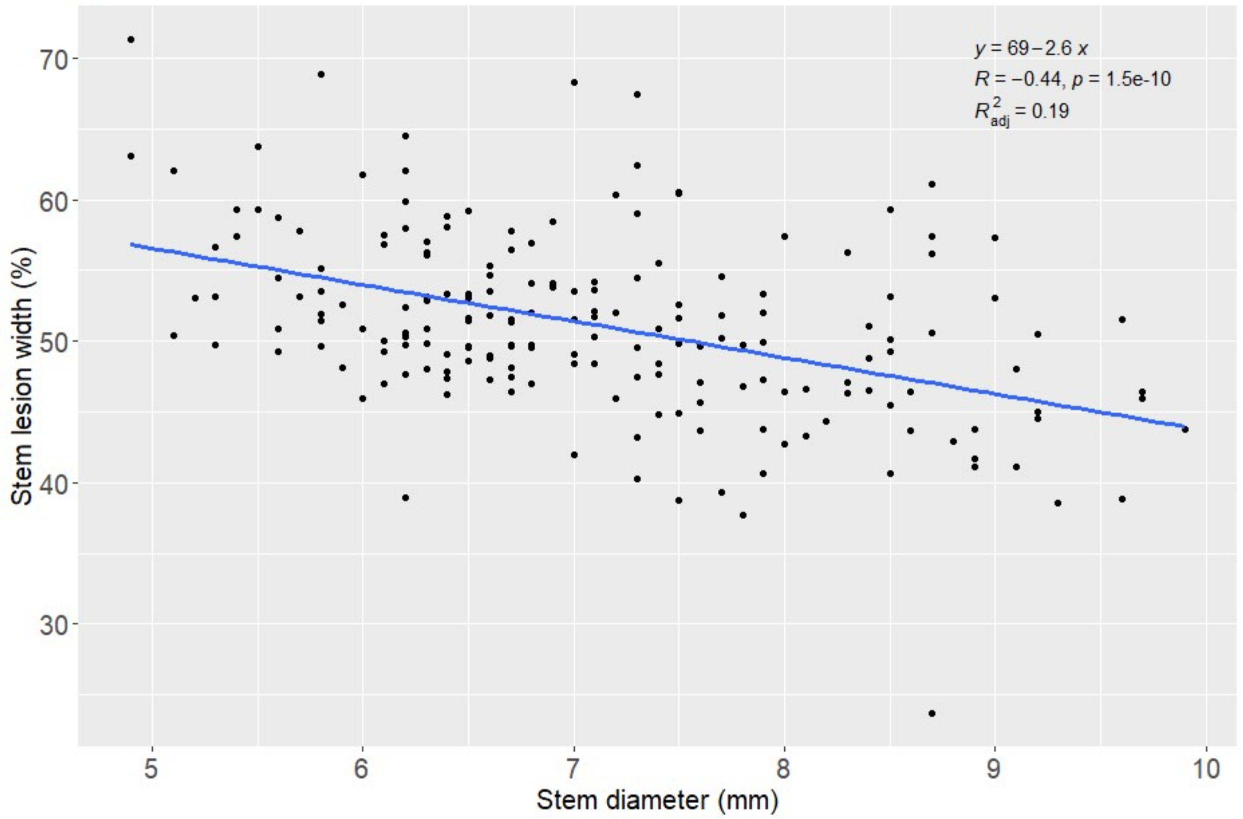

$y = 69 - 2.6\ x$
$R = -0.44,\ p = 1.5e\text{-}10$
$R^2_{adj} = 0.19$
Stem lesion width (%)
Stem diameter (mm)

c

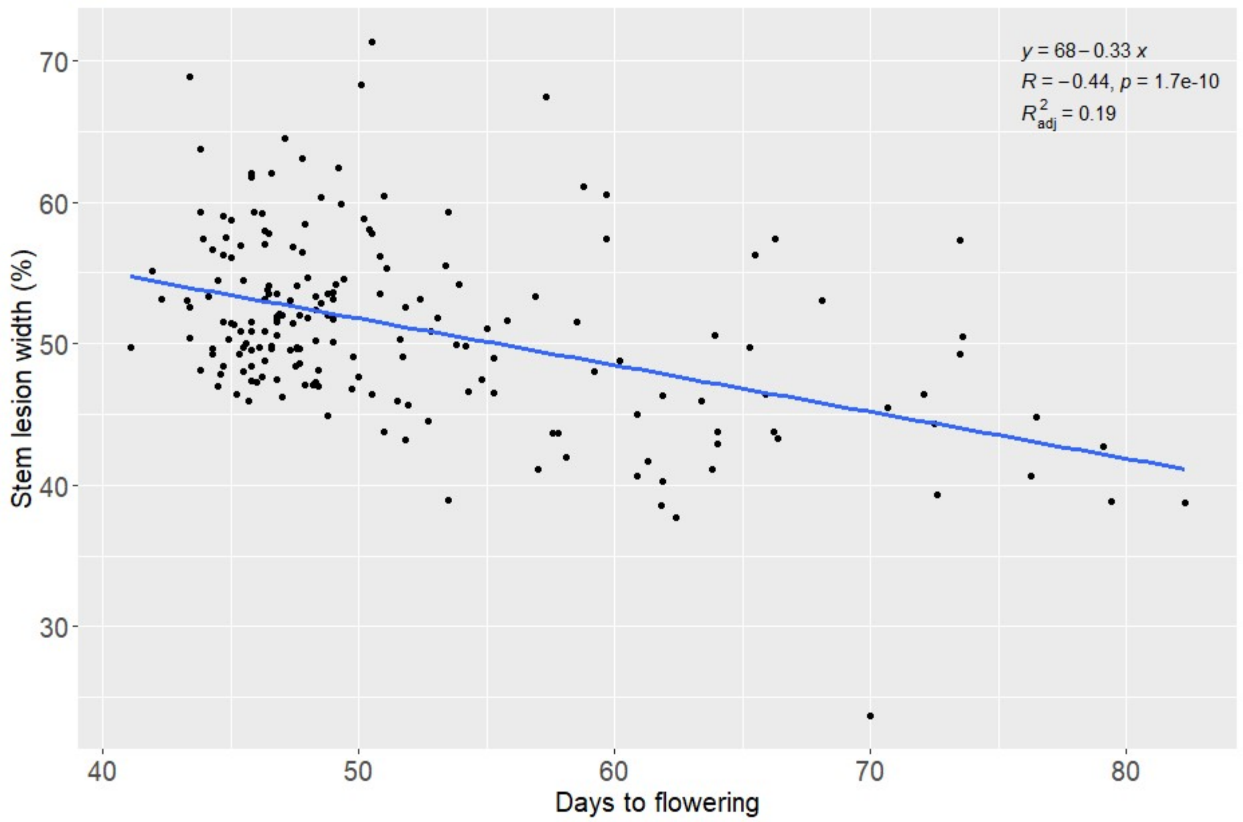
y = 68 − 0.33 x
R = −0.44, p = 1.7e-10
R^2_{adj} = 0.19
Stem lesion width (%)
Days to flowering
70
60
50
40
30
40
50
60
70
80

d

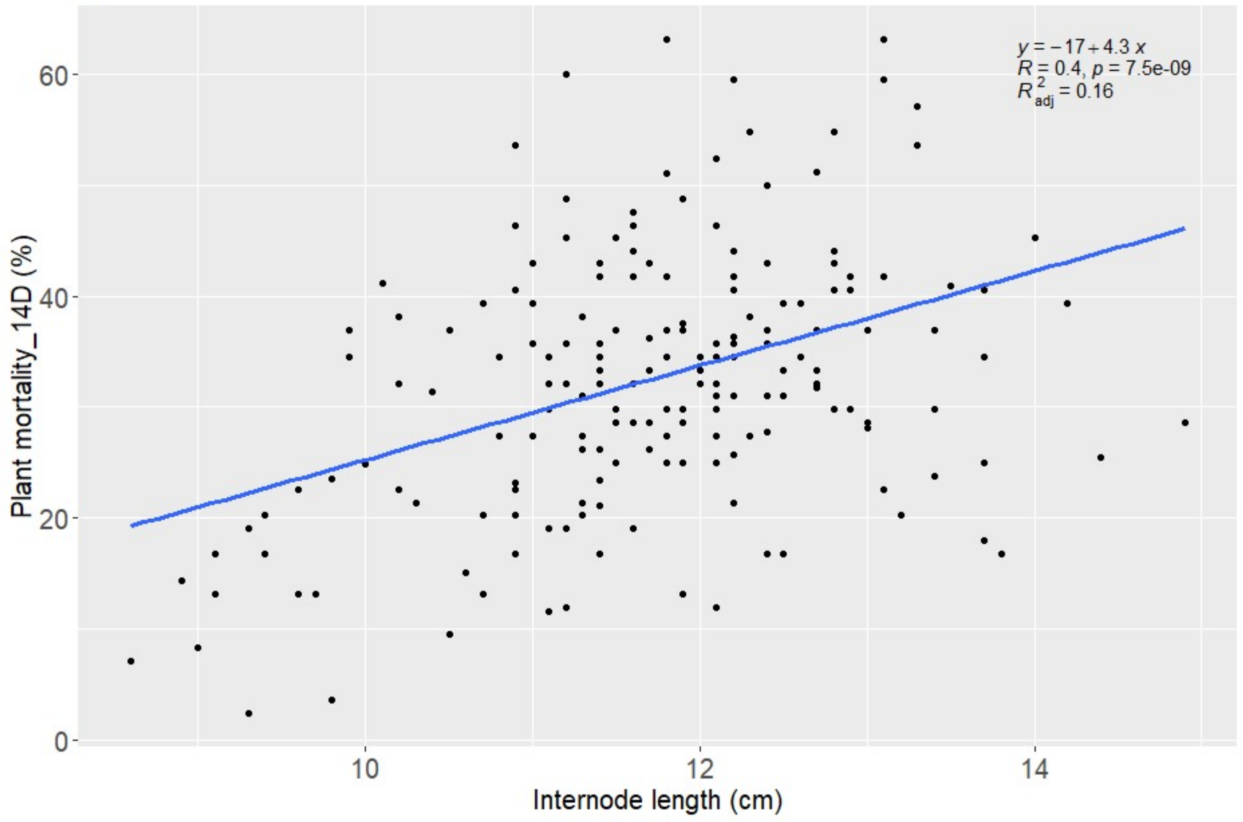
y = −17 + 4.3 x
R = 0.4, p = 7.5e-09
R^2_{adj} = 0.16
Plant mortality_14D (%)
Internode length (cm)
60
40
20
0
10
12
14

e

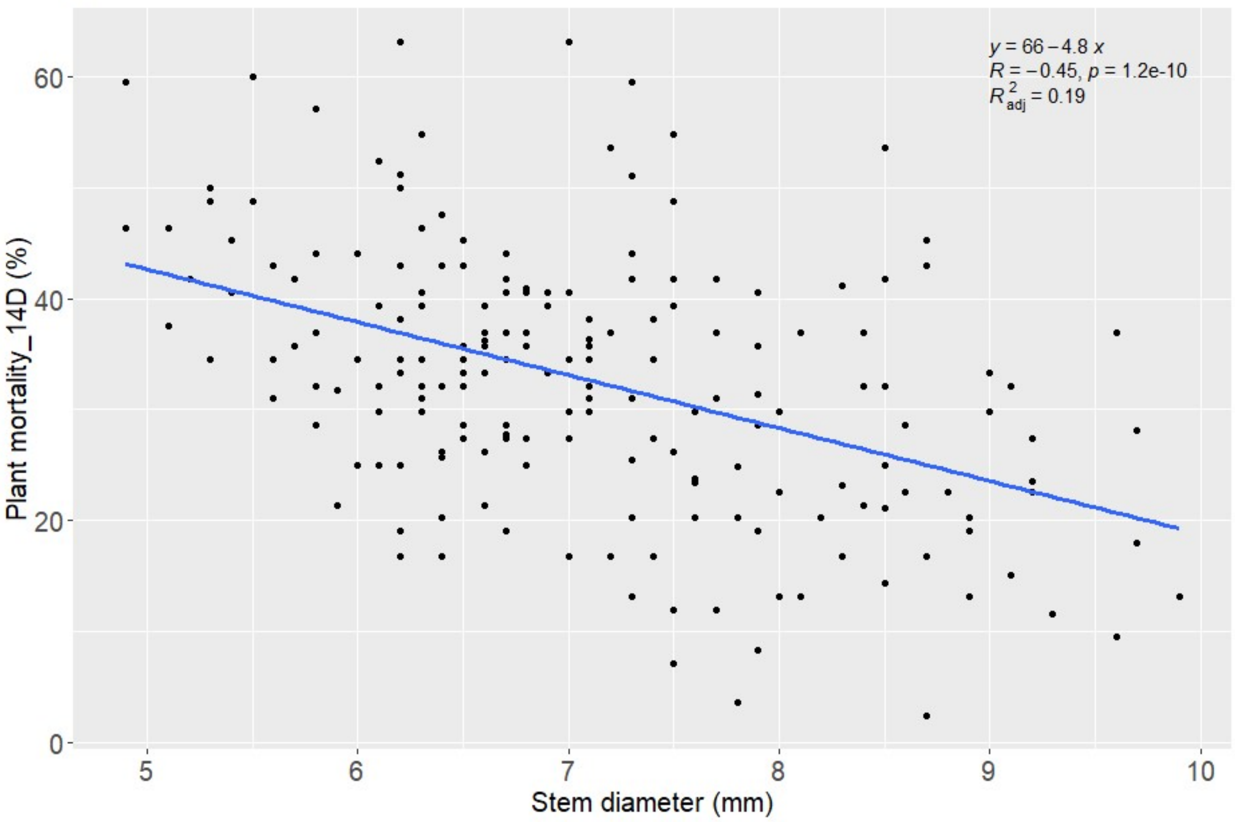
y = 66 − 4.8 x
R = −0.45, p = 1.2e-10
R²adj = 0.19
Plant mortality_14D (%)
Stem diameter (mm)
0
20
40
60
5
6
7
8
9
10

f

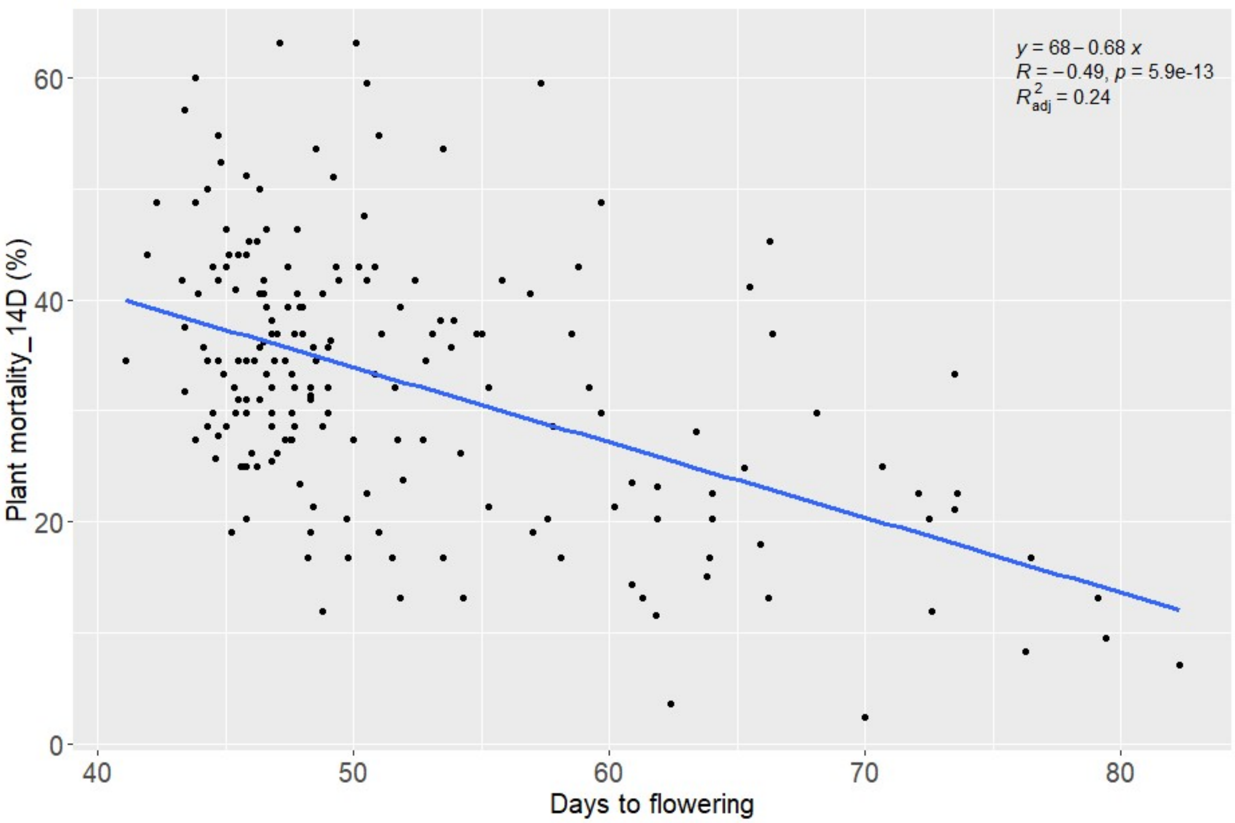
y = 68 − 0.68 x
R = −0.49, p = 5.9e-13
R²adj = 0.24
Plant mortality_14D (%)
Days to flowering
0
20
40
60
40
50
60
70
80

g

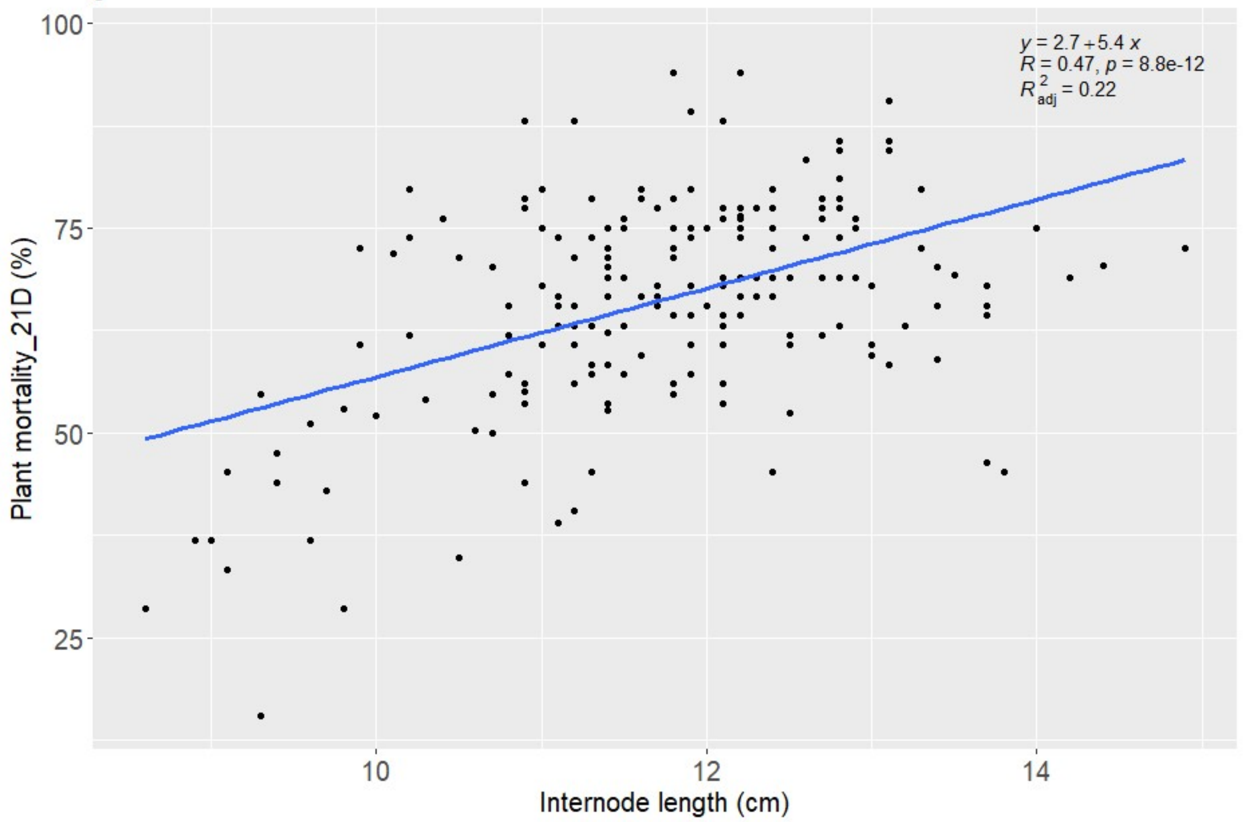
y = 2.7 + 5.4 x
R = 0.47, p = 8.8e-12
R2adj = 0.22
Plant mortality_21D (%)
100
75
50
25
10
12
14
Internode length (cm)

h

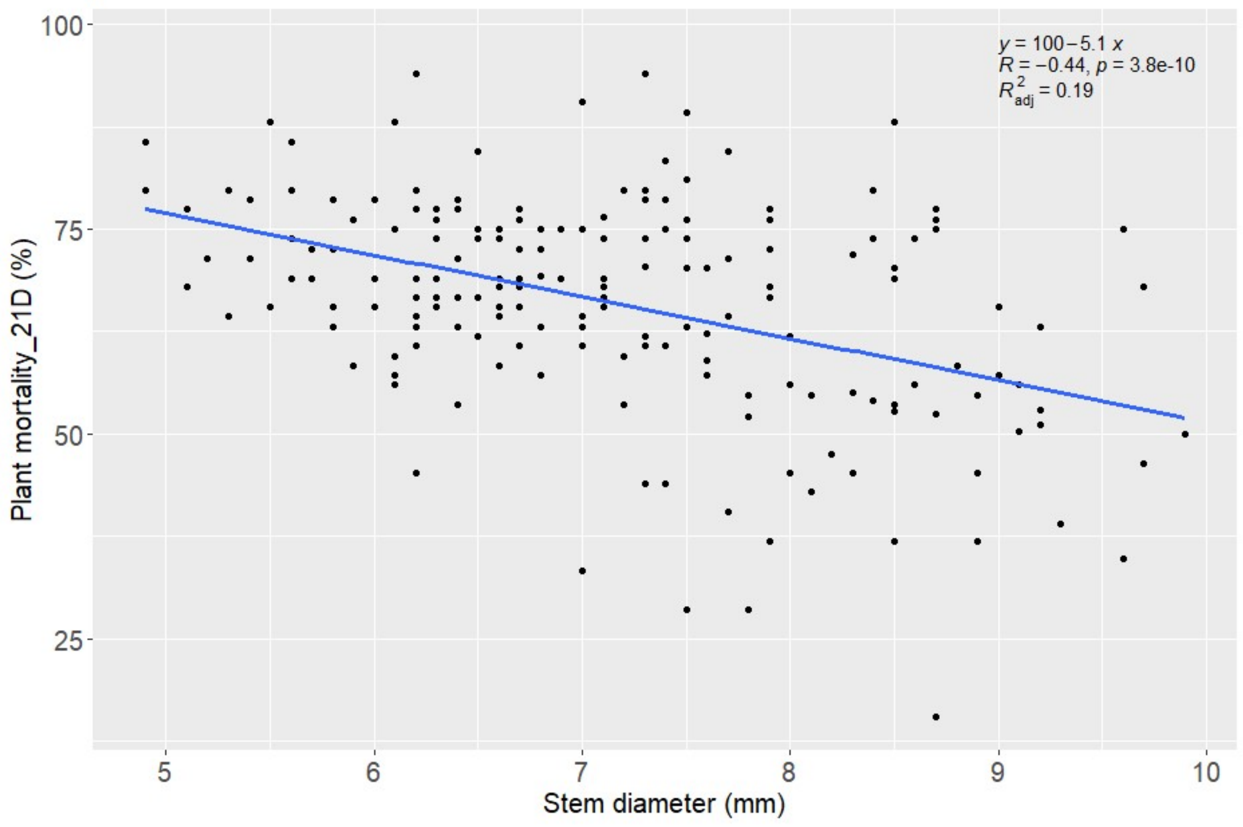
y = 100 − 5.1 x
R = −0.44, p = 3.8e-10
R2adj = 0.19
Plant mortality_21D (%)
100
75
50
25
5
6
7
8
9
10
Stem diameter (mm)

i

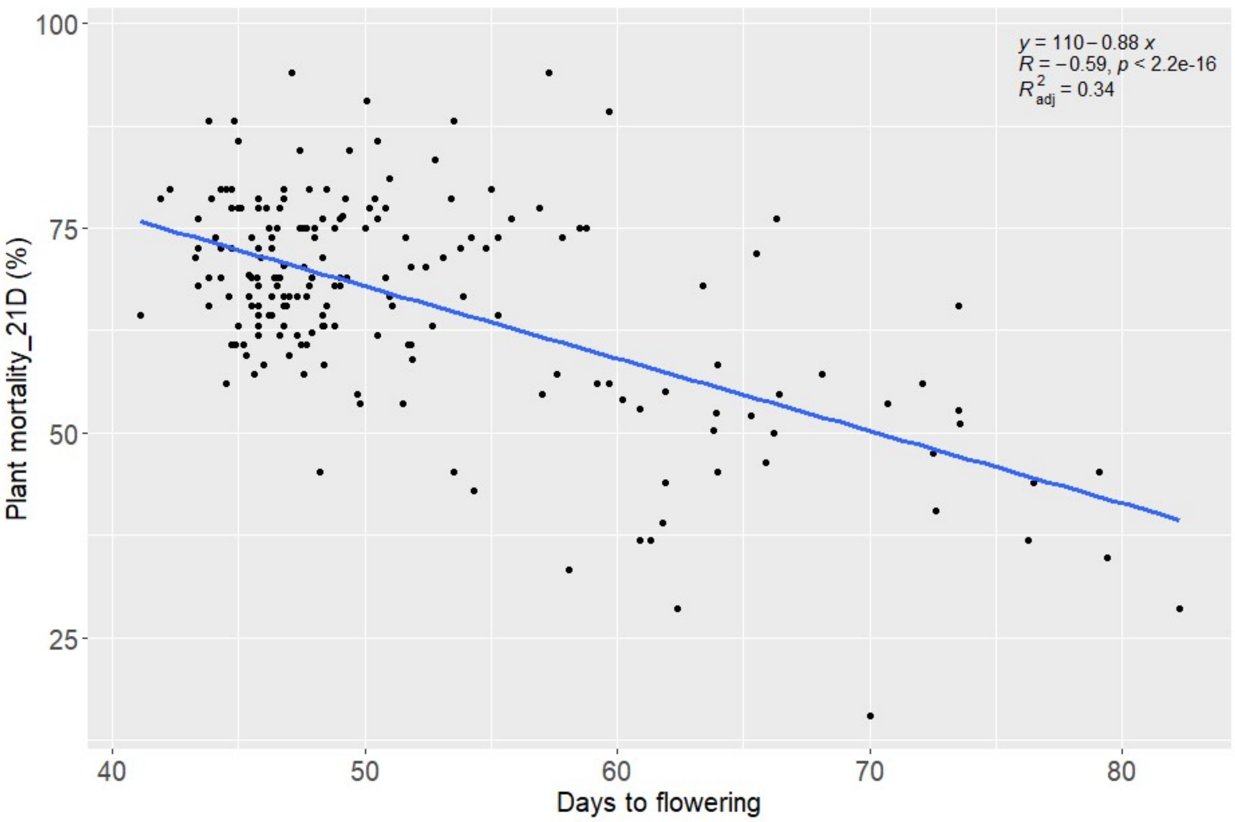

Supplementary Figure S2. Regression analysis of SSR resistance in respect to a) stem lesion width and internode length, b) stem lesion width and stem diameter, c) stem lesion width and days to flowering, d) plant mortality at 14 days post inoculation (PM_14D) and internode length, e) PM_14D and stem diameter, f) PM_14D and days to flowering, g) plant mortality at 21 days post inoculation (PM_21D) and internode length, h) PM_21D and stem diameter, i) PM_21D and days to flowering. *R* is Pearson's correlation coefficient between the two traits, R^2_{adj} is the coefficient of determination.

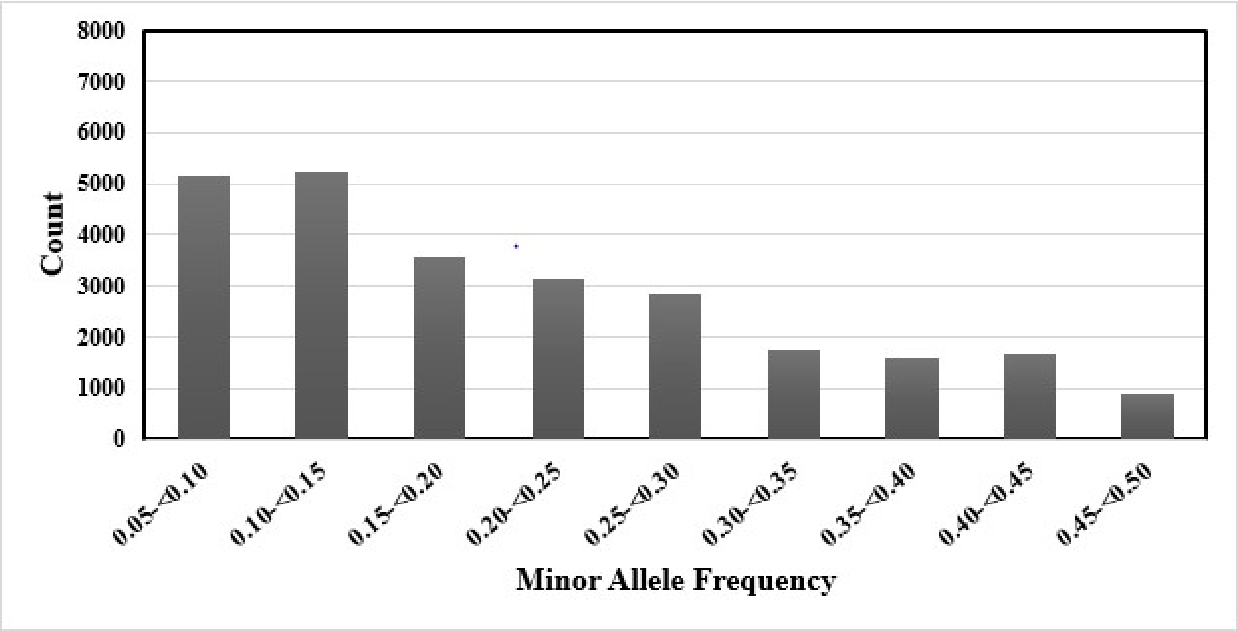

Supplementary Figure S3. Single-nucleotide polymorphism distribution with minor allele frequency in this population panel.

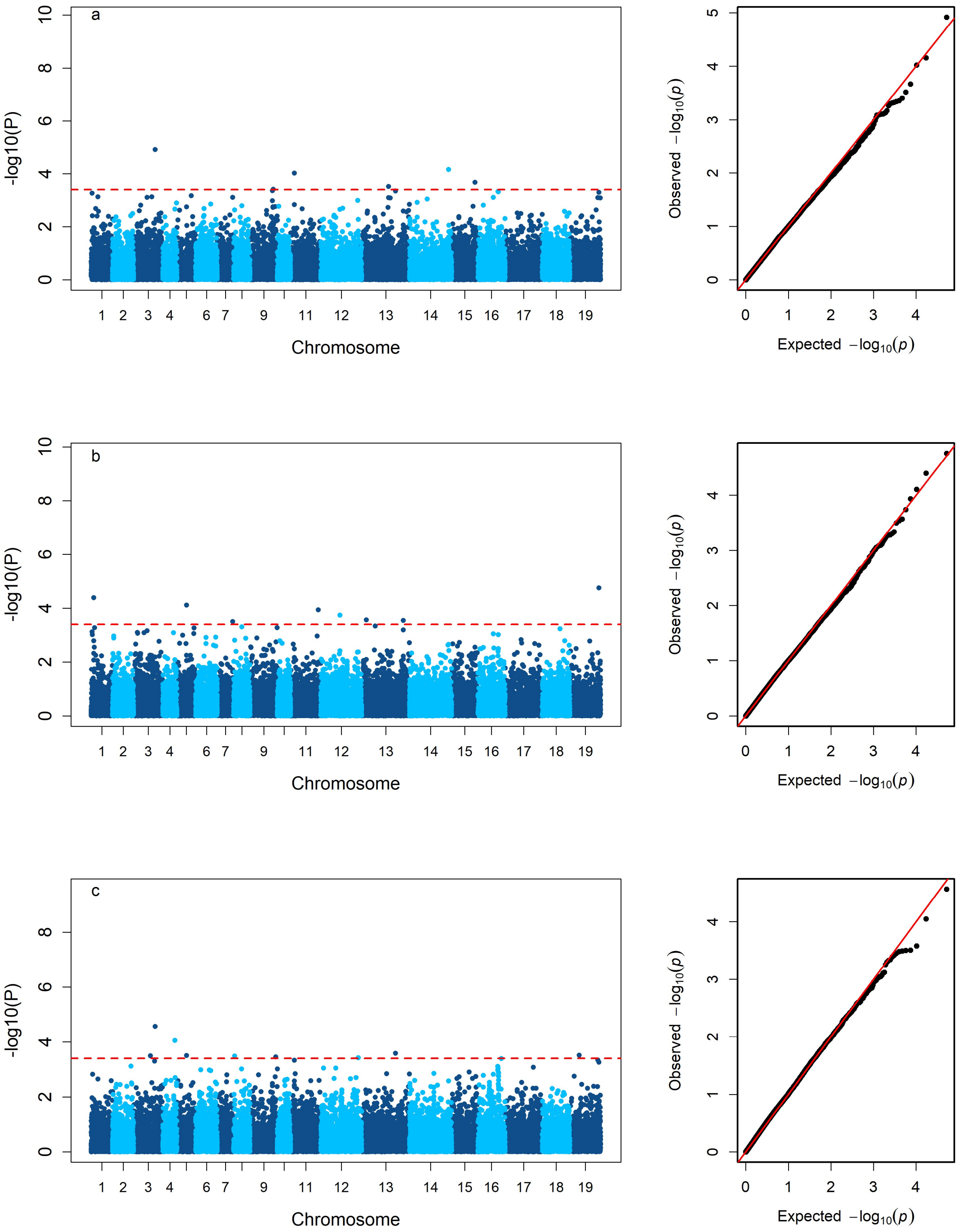
a
b
c
-log10(P)
Chromosome
1 2 3 4 6 7 9 11 12 13 14 15 16 17 18 19
Observed $-\log_{10}(p)$
Expected $-\log_{10}(p)$

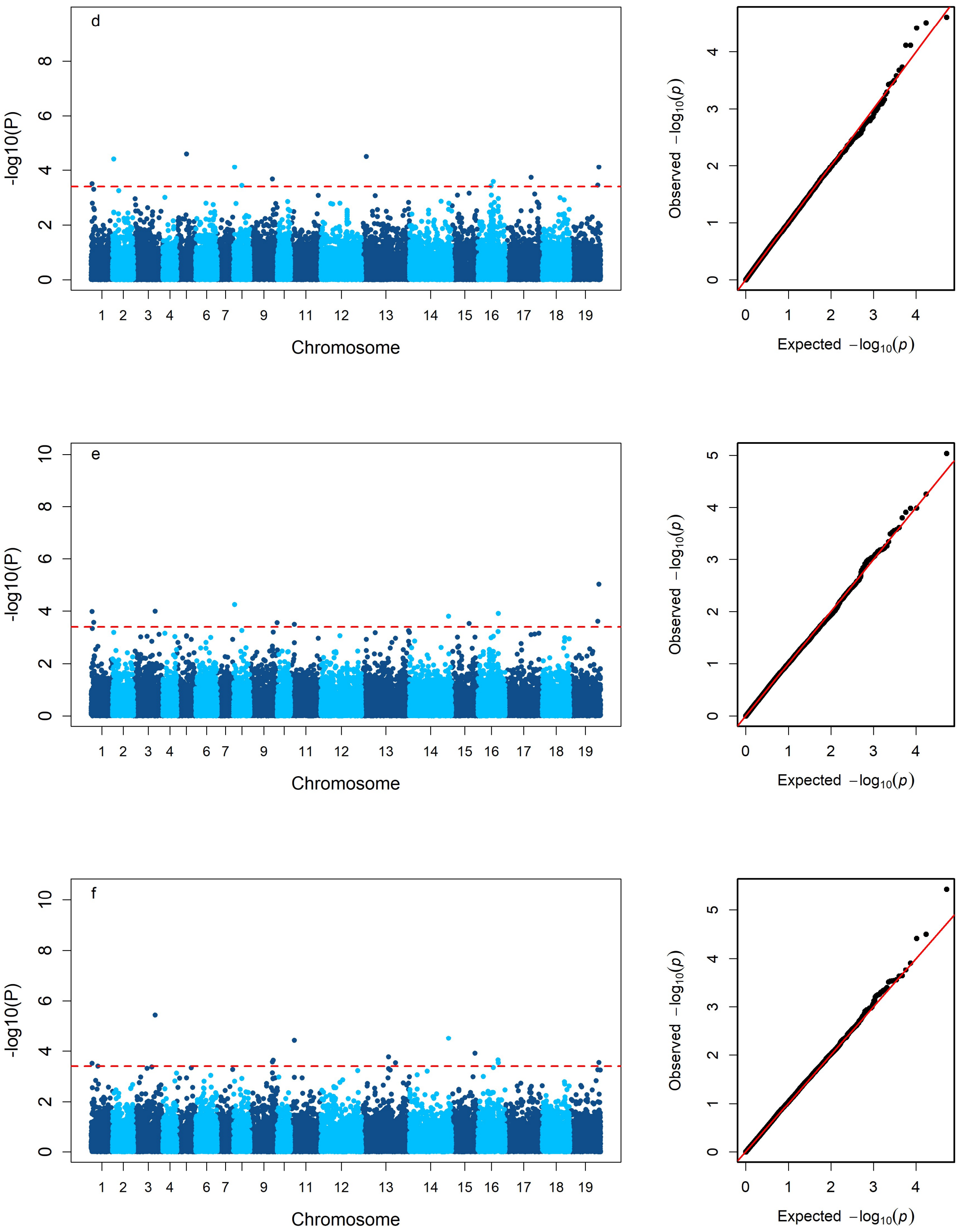

d
e
f
-log10(P)
Chromosome
1 2 3 4 6 7 9 11 12 13 14 15 16 17 18 19
Observed −log10(p)
Expected −log10(p)

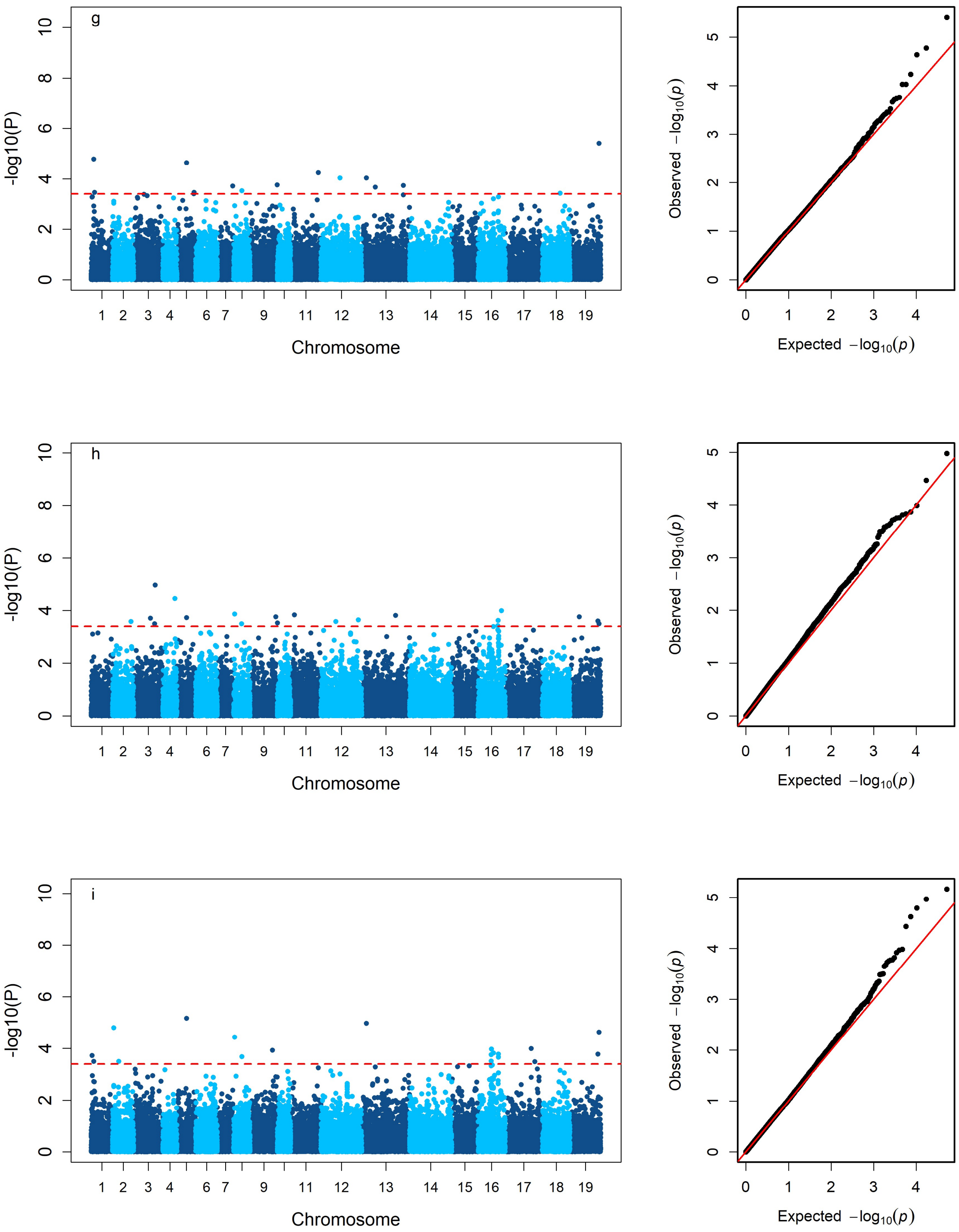
g
h
i
-log10(P)
0 2 4 6 8 10
1 2 3 4 6 7 9 11 12 13 14 15 16 17 18 19
Chromosome
Observed −log10(p)
Expected −log10(p)
0 1 2 3 4 5

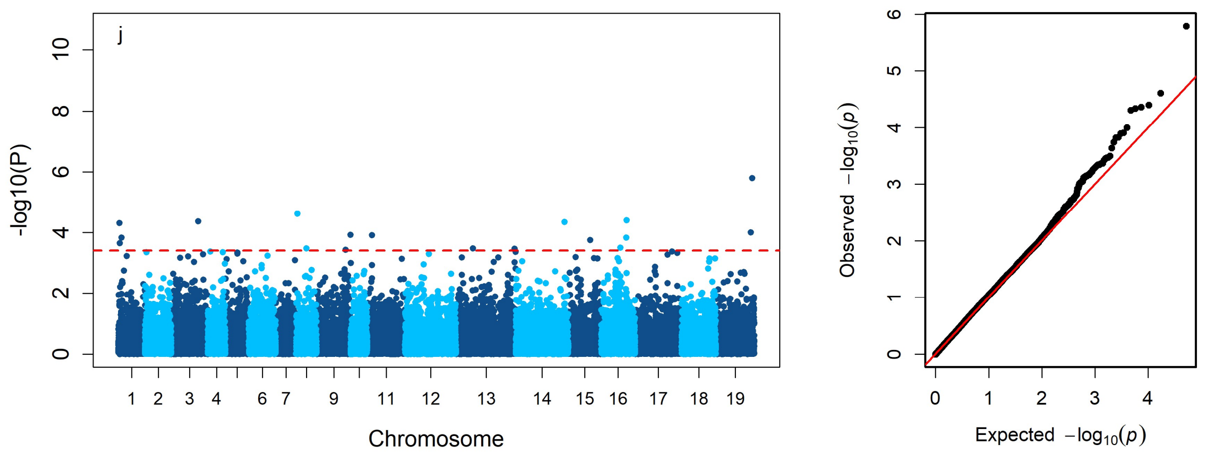

Supplementary Figure S4. Manhattan and Q-Q plots showing the results of marker-trait-associations for stem lesion length associated with sclerotinia stem rot resistance in 187 canola/rapeseed genotypes by the MLM and GEMMA-MLM GWAS model. a) MLM, Carrington 2019; b) MLM, Langdon, 2019; c) MLM, Carrington 2020; d) MLM, Osnabrock 2020; e) MLM, combined data (CombENV), f) GEMMA-MLM, Carrington 2019; g) GEMMA-MLM, Langdon, 2019; h) GEMMA-MLM, Carrington 2020; i) GEMMA-MLM, Osnabrock 2020; j) GEMMA-MLM, combined data (CombENV). The $-\log_{10}$ (P) values from a genome-wide scan are plotted against positions on each of the 19 chromosomes. Discontinued horizontal lines indicate the genome-wide significance threshold.

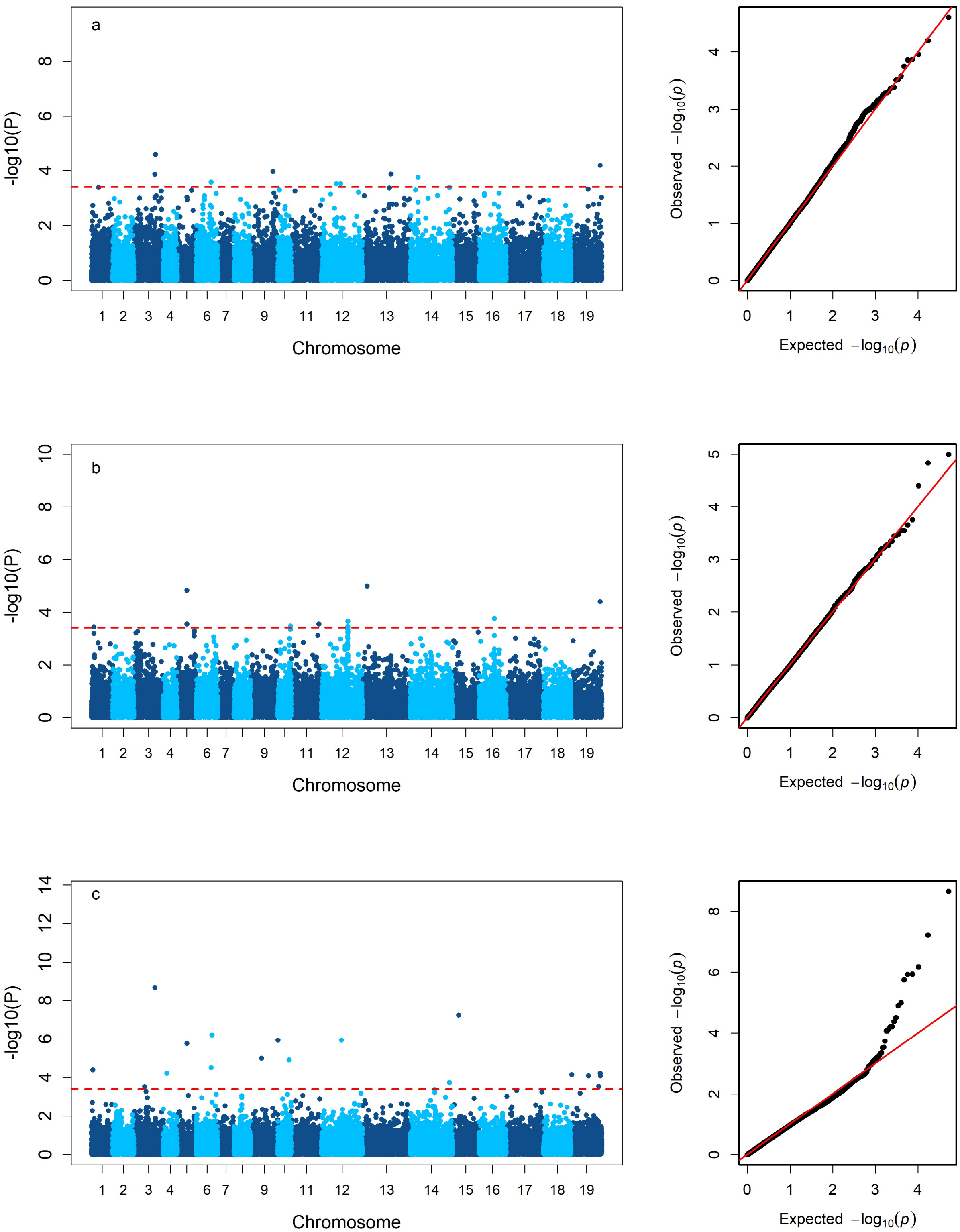
a
b
c
-log10(P)
Chromosome
1 2 3 4 6 7 9 11 12 13 14 15 16 17 18 19
Observed −log10(p)
Expected −log10(p)

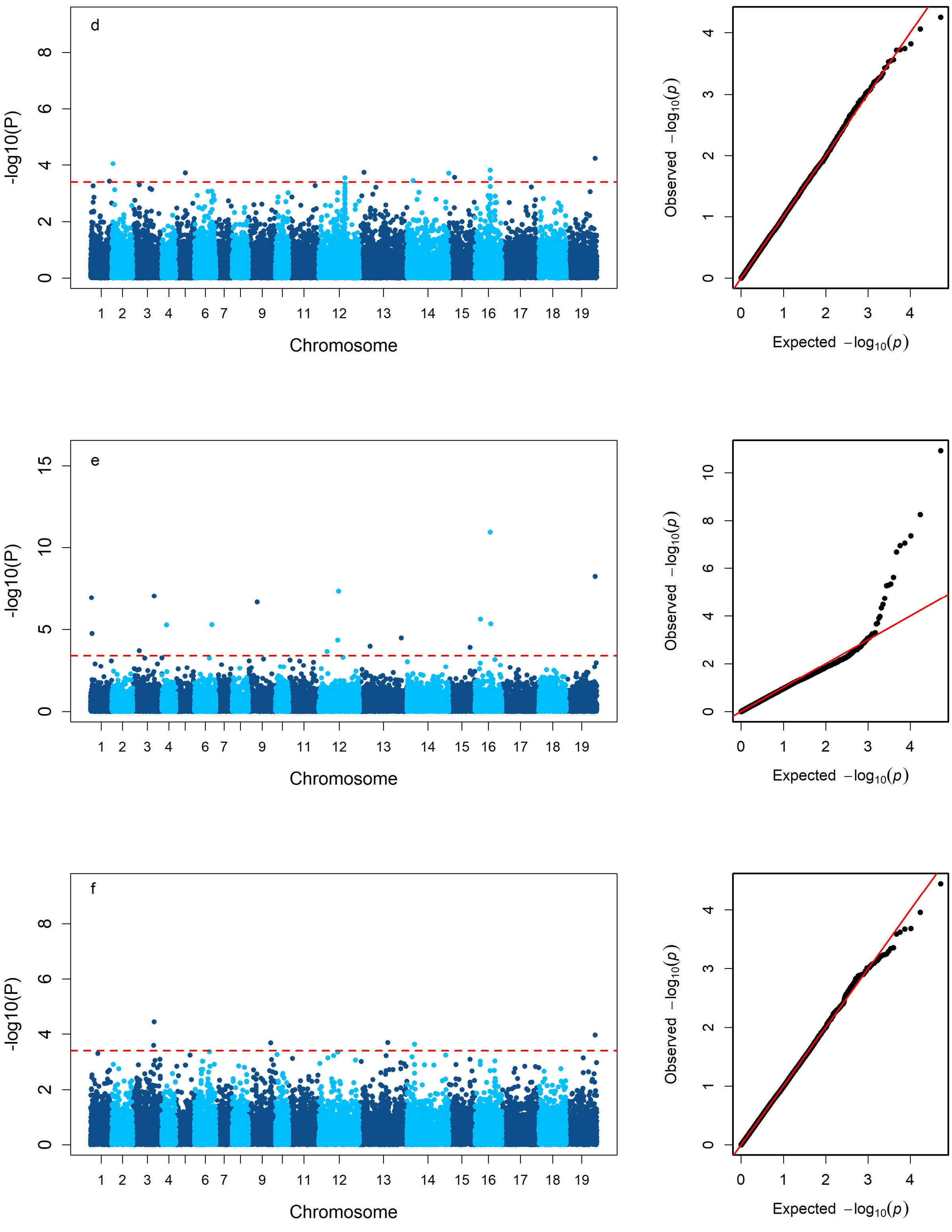
d
-log10(P)
Chromosome
1 2 3 4 6 7 9 11 12 13 14 15 16 17 18 19
Observed $-\log_{10}(p)$
Expected $-\log_{10}(p)$
e
-log10(P)
Chromosome
1 2 3 4 6 7 9 11 12 13 14 15 16 17 18 19
Observed $-\log_{10}(p)$
Expected $-\log_{10}(p)$
f
-log10(P)
Chromosome
1 2 3 4 6 7 9 11 12 13 14 15 16 17 18 19
Observed $-\log_{10}(p)$
Expected $-\log_{10}(p)$

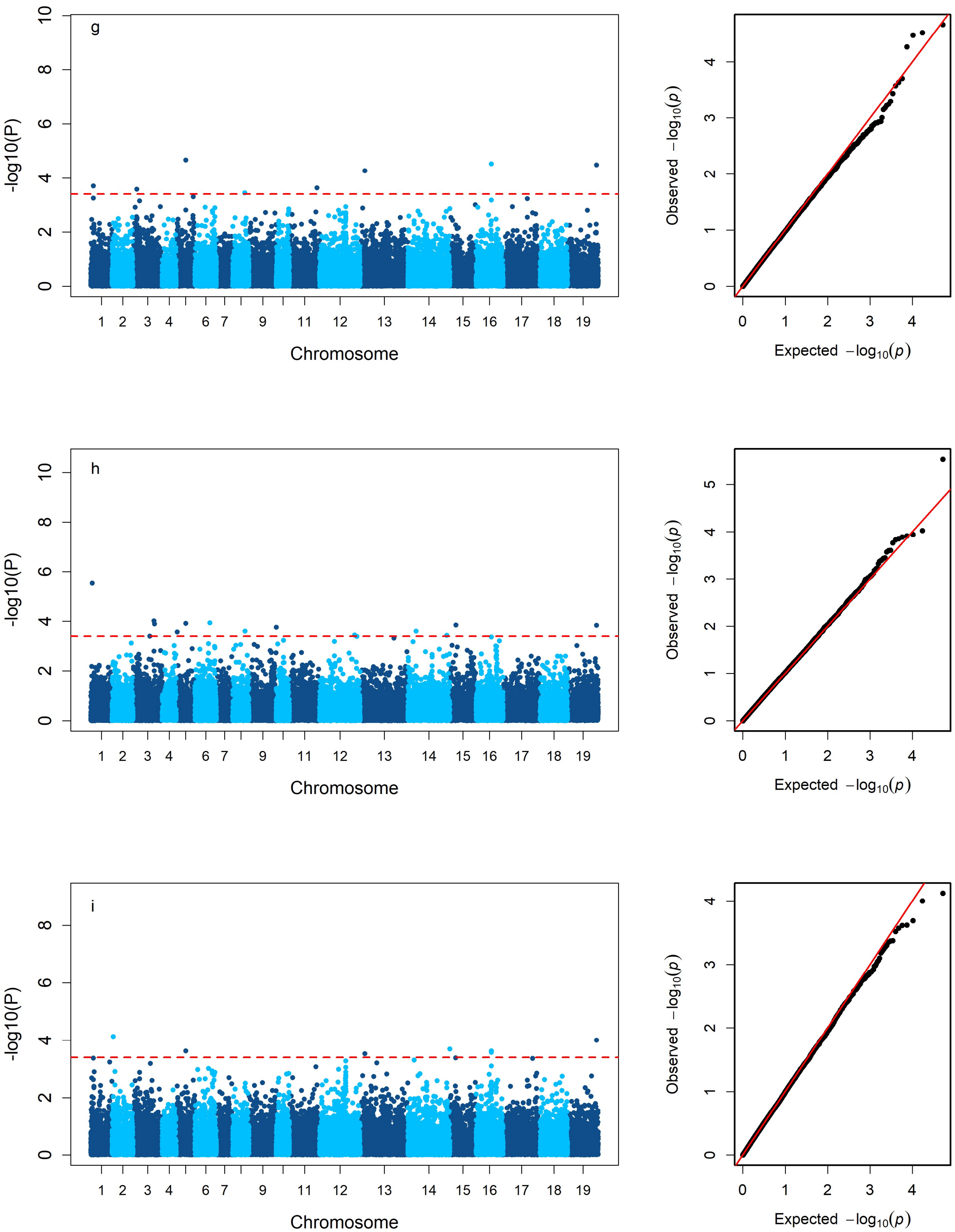
g
h
i
-log10(P)
Chromosome
1 2 3 4 6 7 9 11 12 13 14 15 16 17 18 19
Observed $-\log_{10}(p)$
Expected $-\log_{10}(p)$

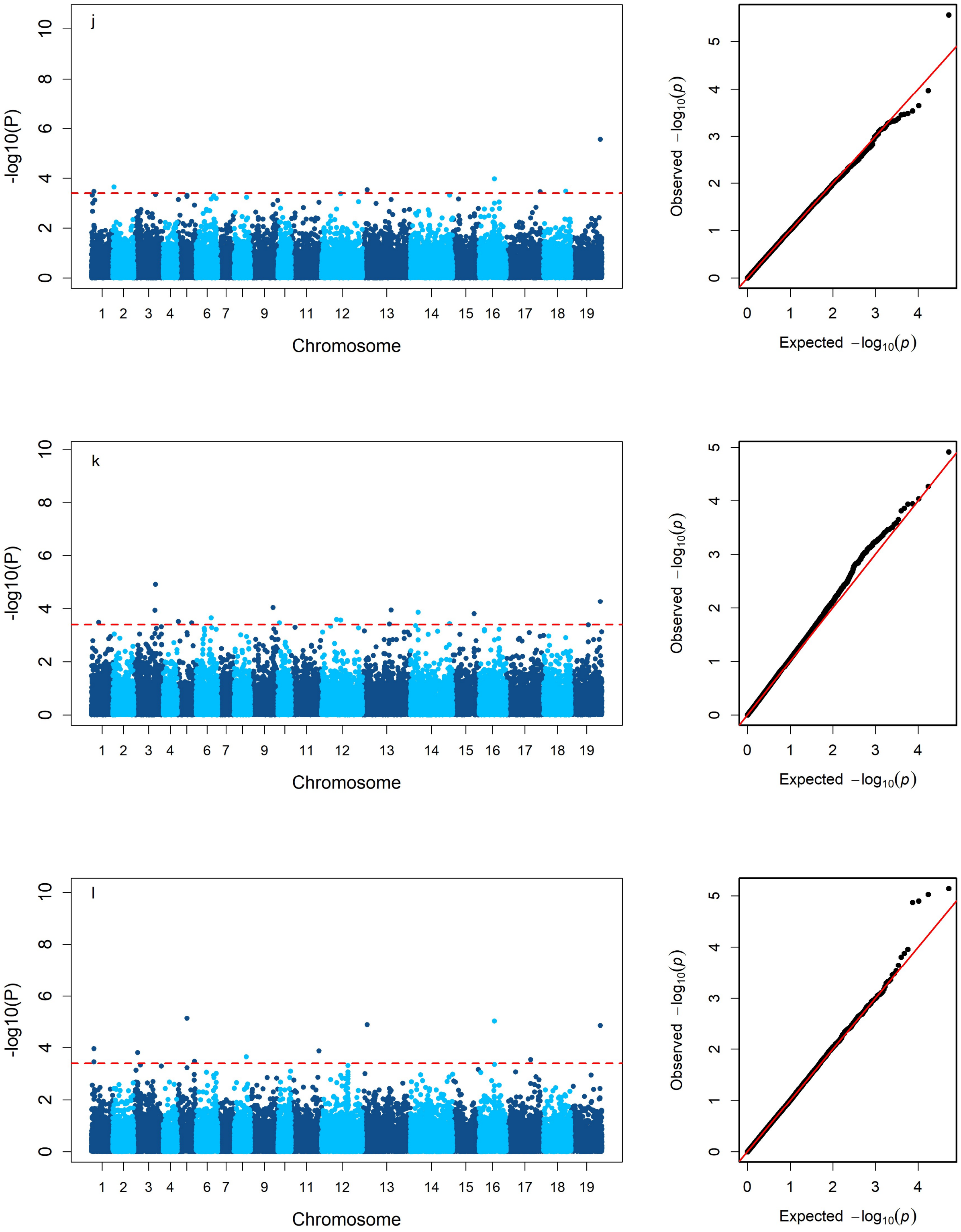
j
k
l
-log10(P)
Chromosome
1 2 3 4 6 7 9 11 12 13 14 15 16 17 18 19
Observed −log10(p)
Expected −log10(p)

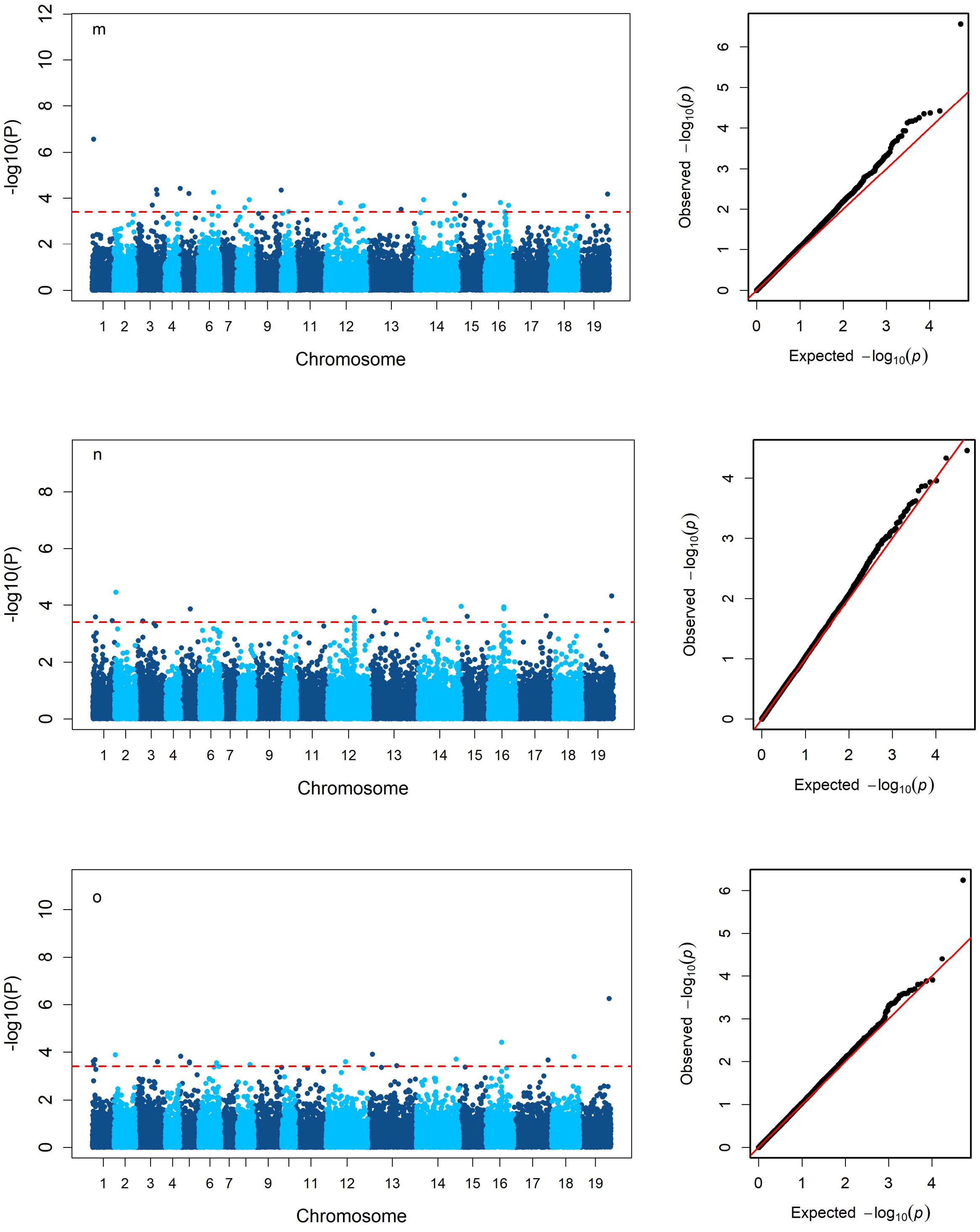

Supplementary Figure S5. Manhattan and Q-Q plots showing the results of MTAs for stem lesion width associated with SSR resistance in 187 canola/rapeseed genotypes by the FarmCPU and MLM GWAS model. a) FarmCPU, Carrington 2019; b) FarmCPU, Langdon, 2019; c) FarmCPU, Carrington 2020; d) FarmCPU, Osnabrock 2020; e) FarmCPU, combined data (CombENV), f) MLM, Carrington 2019; g) MLM, Langdon, 2019; h) MLM, Carrington 2020; i) MLM, Osnabrock 2020; j) MLM, combined data (CombENV); k) GEMMA-MLM, Carrington 2019; l) GEMMA-MLM, Langdon, 2019; m) GEMMA-MLM, Carrington 2020; n) GEMMA-MLM, Osnabrock 2020; o) GEMMA-MLM, combined data (CombENV). The -log$_{10}$ (*P*) values from a genome-wide scan are plotted against positions on each of the 19 chromosomes. Discontinued horizontal lines indicate the genome-wide significance threshold.

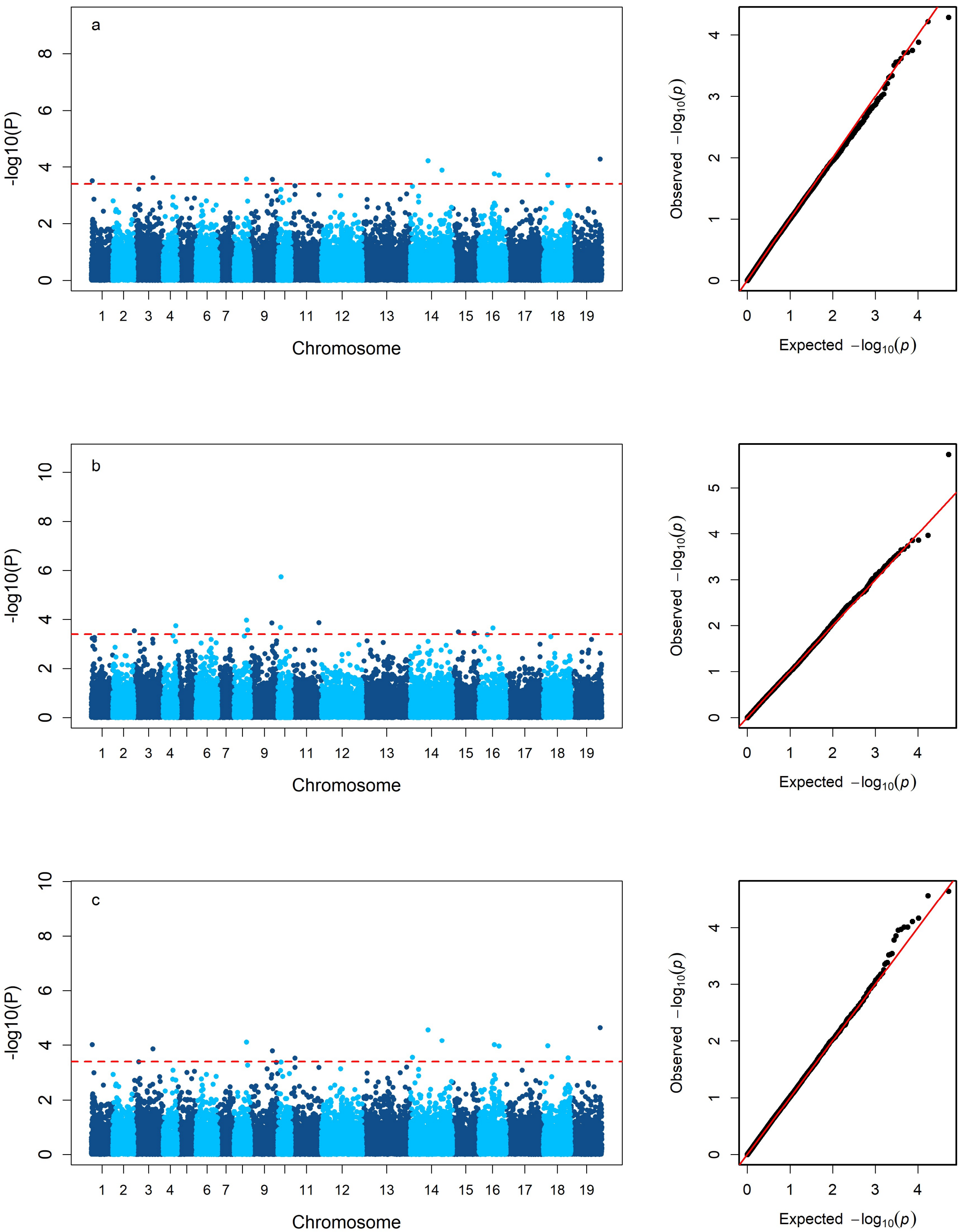

a
b
c
-log10(P)
Chromosome
1 2 3 4 6 7 9 11 12 13 14 15 16 17 18 19
Observed −log10(p)
Expected −log10(p)

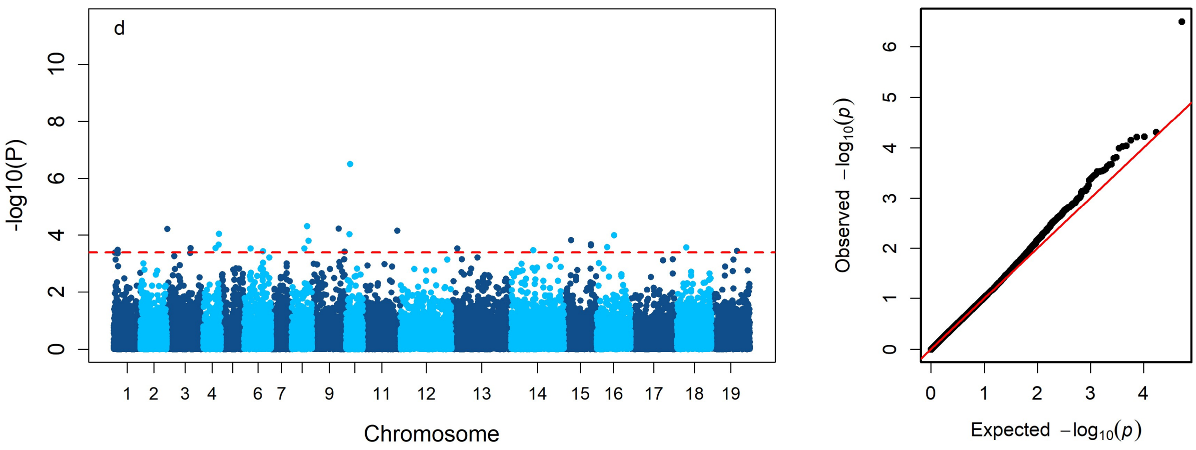

Supplementary Figure S6. Manhattan and Q-Q plots showing the results of marker-trait association for plant mortality associated with sclerotinia stem rot resistance in 187 canola/rapeseed genotypes by the MLM and GEMMA-MLM GWAS model. a) MLM, combined (CombENV) plant mortality at 14 days post inoculation (dpi); b) MLM, combined (CombENV) plant mortality at 21 dpi; c) GEMMA-MLM, combined (CombENV) plant mortality at 14 dpi; d) GEMMA-MLM, combined (CombENV) plant mortality at 21 dpi. The $-\log_{10}$ (P) values from a genome-wide scan are plotted against positions on each of the 19 chromosomes. Discontinued horizontal lines indicate the genome-wide significance threshold.

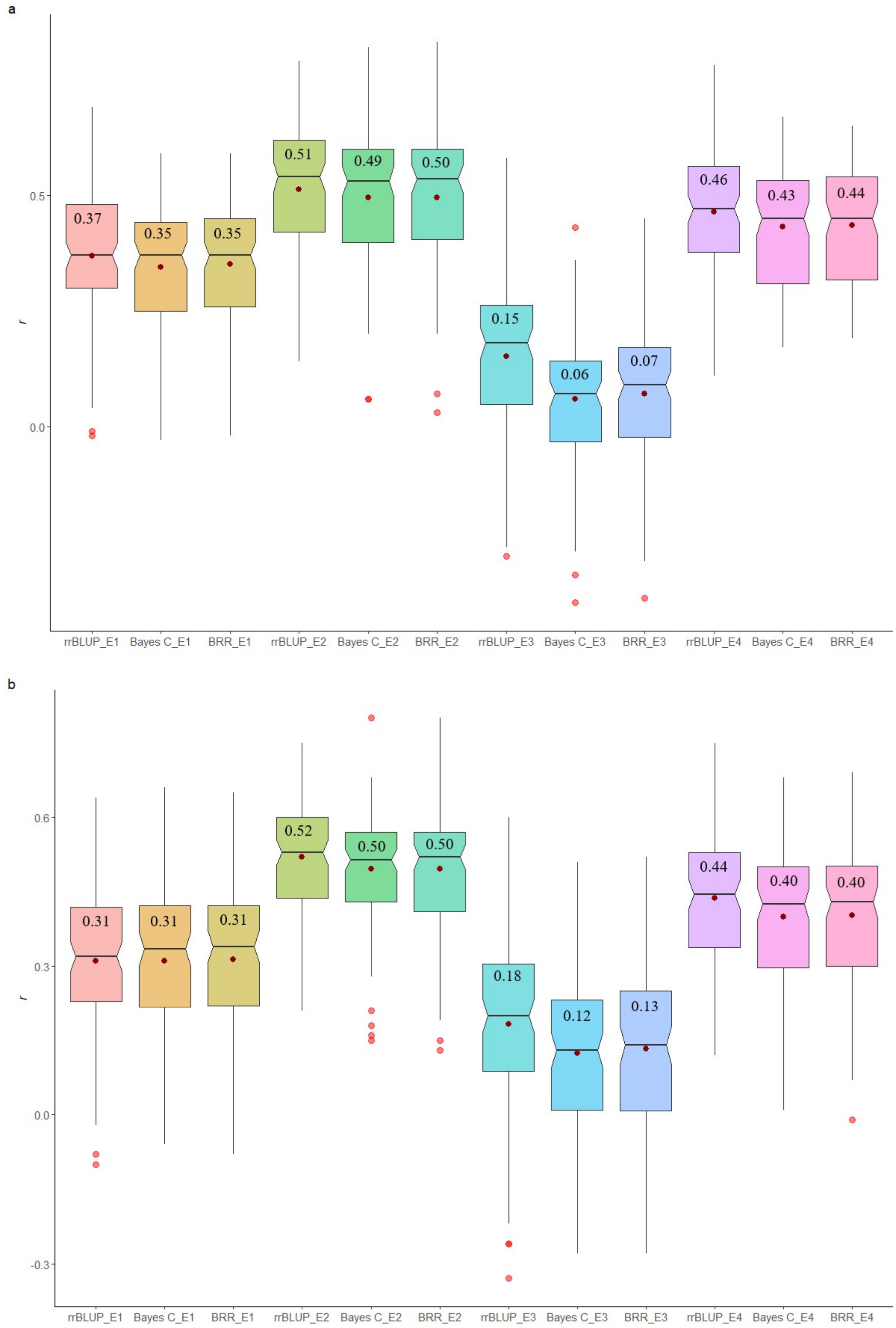

Supplementary Figure S7. Predictive ability for sclerotinia stem rot resistance estimated from the five-fold cross-validation schemes of the association panel. a) Boxplot showing the result of average predictive ability (*r*) (y axis) for lesion length, b) lesion width in four environments with rrBLUP, Bayes C and Bayesisan ridge regression (BRR) models (x-axis). E1, E2, E3, and E4 represents Carrington 2019; Langdon 2019; Carrington 2020; Osnabrock 2020 environements respectively. The red dot in each box plot represent the mean predictive ability. The boxes show second and third quartiles and wishkers show interquertile range. The number above horizontal black bars are the predictive ability (*r*).
